# Perceived stigma among hospitalized pulmonary tuberculosis patients in Hue Central Hospital: A 2024 mixed method study in Vietnam

**DOI:** 10.1371/journal.pgph.0006752

**Published:** 2026-07-13

**Authors:** Thao Thi Van Luong, Huong Thi Thanh Le, Anh Quynh Nguyen, Bao Quy Quoc Truong, Thuy Thi Thu Tran

**Affiliations:** 1 General Planning Department, Hue Central Hospital, Hue, Vietnam; 2 Faculty of Environmental and Occupational Health, Hanoi University of Public Health, Hanoi, Vietnam; 3 Faculty of Public Health, Hue University of Medicine and Pharmacy, Hue University, Hue, Vietnam; University of Sydney, AUSTRALIA

## Abstract

Perceived tuberculosis related stigma remains a critical structural barrier to timely healthcare-seeking and a major driver of the global tuberculosis burden. This study aimed to estimate the prevalence of perceived stigma and identify its associated factors among hospitalized pulmonary tuberculosis inpatients in Hue Central Hospital - a national tertiary hospital in Vietnam - in 2024. A mixed-methods design was employed, incorporating both quantitative and qualitative components. The quantitative component utilized the validated Van Rie Tuberculosis Stigma Scale (VTSS) with 200 pulmonary tuberculosis inpatients from May to July 2024 to measure multiple dimensions of perceived stigma. Additionally, 13 in-depth interviews were conducted with inpatients and healthcare workers following the quantitative phase. Quantitative data were analyzed using multivariable regression while qualitative data were audio-recorded, transcribed, thematically coded, and used to enrich interpretation of quantitative findings. Overall, 43.5% of inpatients experienced perceived stigma, 90.5% intentionally kept physical distance from others, and 87.0% disclosed their disease status selectively to close family members. Perceived stigma was more prevalent among patients with higher socioeconomic status (aOR = 2.01; 95%CI: 1.01-3.99), and those who were unmarried or widowed (aOR = 2.93; 95%CI: 1.01-8.47). Qualitative results further highlighted how social isolation, fear of contagion, and persistent cultural misconceptions about tuberculosis - compounded by the visibility of a tertiary hospital - significantly shaped these perceived stigmatizing experiences. Perceived pulmonary tuberculosis related stigma among pulmonary tuberculosis inpatients remains high. These findings underscore the urgent need for integrating stigma-reduction strategies into routine tuberculosis programs, including community education, patient-centered counselling, and strengthened social support mechanisms to mitigate the psychological burden of the disease.

## Introduction

Tuberculosis (TB) remains a leading global health threat [1], with approximately 10.7 million new cases and 1.23 million deaths reported in 2024 [2]. Vietnam is currently ranked among the top 30 countries for TB and multidrug-resistant TB (MDR-TB) burden [2]. Recent studies emphasize that structural health system disruptions and diagnostic inequalities significantly influence care pathways [3]. In Vietnam, barriers to accessing public diagnostic services - including geographical distance and administrative complexities - continue to impede early detection [4]. Furthermore, efforts to integrate TB patients into social health insurance highlight the ongoing challenges in achieving universal health coverage for vulnerable populations [5].

A critical structural barrier undermining these control efforts is stigma. Conceptually, TB-related stigma is a multi-dimensional construct involving enacted, anticipated, and internalized stigma [6,7]. Within this framework, perceived stigma (also referred to as felt stigma) represents an essential psychological dimension, defined as an individual’s internal awareness of negative social attitudes and the fear of encountering discrimination, even in the absence of direct discriminatory behaviors [7]. This perception is often categorized into anticipated stigma - the fear of being socially devalued if one’s status is revealed - and internalized stigma, where patients adopt negative social conceptions as their own, leading to profound feelings of shame [8].

In Vietnam, recent evidence has revealed alarmingly high rates of perceived stigma among TB patients. Research by Redwood et al. (2022) indicated that nearly all participants experienced some form of perceived stigma, with profound concerns over ‘family reputation’ and ‘social prestige’ leading to self-isolation [9]. More specifically, among patients with drug-resistant TB, Huy Le Ngoc et al. (2026) reported significant levels of perceived stigma, with mean Van Rie Stigma scores reflecting a persistent ‘post-cure stigma paradox’ where patients continue to feel socially devalued even after clinical recovery [8]. These findings suggest that for Vietnamese patients, the fear of social status loss often outweighs the perceived benefits of early clinical engagement [8,9].

Perceived stigma initiates a reinforcing cycle that exacerbates pulmonary TB (pTB) outcomes by driving concealment and delaying healthcare-seeking, thereby sustaining community transmission [10]. Beyond social and familial domains, institutional factors remain a significant concern, for instance, the attitudes of healthcare professionals can inadvertently reinforce a patient’s perceived stigma, further discouraging adherence to treatment protocols [11]. Effective interventions, therefore, require a clear understanding of the conceptual pathways linking these internal perceptions to clinical outcomes [10,12].

To effectively quantify these subjective experiences, the Van Rie TB Stigma Scale (VTSS) has been validated as a robust tool in the Vietnamese clinical setting [13], specifically designed to capture the dimensions of patient-centered perceived stigma [8,13].

The Department of Pulmonary Diseases of Hue Central Hospital serves as is a leading tertiary referral center for diagnosis and treatment of TB in the Central and Central Highlands regions of Vietnam. The Department manages a high number of complex TB cases. In 2024 alone, the hospital’s Department of Pulmonary Diseases recorded 2,849 consultations and performed 3,698 sputum examinations. During the same period, the hospital managed 1,691 pTB admissions, while 378 cases were referred to lower-level facilities for management under the Directly Observed Treatment Short-course (DOTS) program. An average of 15–20 pTB patients were hospitalized each week, with a mean length of stay of approximately 15.5 days [14]. This high-density clinical environment, where patients from diverse provinces seek specialized care, provides a unique lens to examine how structural health barriers and the visibility of a national institution intersect with cultural perceptions of stigma.

This study addresses a comprehensive research question: What is the prevalence of perceived TB-related stigma among inpatients at a national tertiary hospital in Vietnam, and how do socioeconomic factors, cultural perceptions of prestige, and structural healthcare barriers collectively shape these stigmatizing experiences? Using a mixed-methods approach, we aim to assess the prevalence of perceived stigma and identify its associated factors among pTB inpatients at Hue Central Hospital in 2024.

## Materials and methods

### Study design, time, and location

The study employed a mixed method research design, combining both quantitative and qualitative approaches. The quantitative component was conducted first to determine the level of perceived stigma among the study participants. After that, the qualitative component was implemented to identify factors related to patients’ perceived stigma and to provide explanation for the quantitative findings.

The study was undertaken at the Department of Pulmonary Diseases, Hue Central Hospital, Hue City, Vietnam from June 2023 to November 2024 and the data collection period took place from May 25, 2024 to July 15, 2024.

Established in 1894, Hue Central Hospital is the largest tertiary-level national hospital in the Central of Vietnam, serving as a major referral hub for the Central and Highland regions [15]. This setting was selected because it provides a unique clinical environment where patients with complex, multidrug-resistant, or severe pulmonary TB are treated. The facility is equipped with specialized isolation wards, advanced diagnostic laboratories (including GeneXpert and liquid culture systems), and a multidisciplinary team of specialists. Unlike local TB units, Hue Central Hospital manages a diverse patient population from multiple provinces, offering a broader perspective on the systemic and structural barriers faced by patients navigating the highest tier of the national healthcare system [15].

### Study participants

For the quantitative component of the study, participants were recruited among inpatients with pTB at the Department of Pulmonary Diseases of Hue Central Hospital. Eligible patients were those aged 18 years or older who provided informed consent to participate. Patients who were illiterate, acquired HIV, or had documented mental disorders were excluded from participation.

For the qualitative component, participants were purposely selected from the two groups. The first group included pTB patients who had joined the quantitative study. The second group comprised healthcare providers, including doctors and nurses, who had worked for more than three months in the Department of Pulmonary Diseases of Hue Central Hospital, directly involved in clinical care for pTB patients, and agreed to participate in the study.

### Sample size and selection of participants

The quantitative component of the study employed a formula for estimating a population proportion to calculate the minimum required sample size of pTB patients. With confidence level (%) at 95%, an absolute precision of 0.07, and an anticipated prevalence of self-reported stigma among pTB patients of 56% - as reported in a previous study conducted in Cambodia using the Van Rie’s TB stigma scale (VTSS) [16], the required minimum sample size was 193 participants with pTB.

For the quantitative component, we employed a consecutive sampling approach. Specifically, all eligible pTB inpatients admitted to the Department of Pulmonary Disease at Hue Central Hospital during the data collection period (May 25 to July 15, 2024) were approached and invited to participate until the target sample size was reached.

For the qualitative component, we used purposive sampling to select participants from two groups: (1) pTB patients who had participated in the quantitative survey (with variation in stigma experiences), and (2) healthcare providers of department levels. The qualitative component included 13 in-depth interviews, including one of Head of Department of Pulmonary Diseases, three doctors, and three nurses working in the Department of Pulmonary Diseases, with six pTB patients whose level of perceived stigma had been determined through the quantitative study.

### Outcome variable

The main outcome variable of this study was the level of perceived stigma among pTB inpatients, assessed using the Vietnamese validated version of the Van Rie Tuberculosis Stigma Scale (VTSS) [13]. Following the validation process, the Vietnamese version comprises nine items - compared with 12 items in the original scale – capturing the emotional, social, and behavioral aspects of stigma experienced by individuals diagnosed with TB. Items were also changed from third person to first person. Each item was rated on a 4-point Likert scale, ranging from 0 (Strongly disagree) to 3 (Strongly agree) [13]. The total VTSS score, calculated as the sum of scores across all nine items, ranges from 0 to 27, with higher scores reflecting greater levels of perceived stigma. The Cronbach’s alpha coefficient of VTSS in this study sample was 0.82, indicating good reliability.

A raw summary score (SSraw) was calculated by summing responses across all items. These raw scores were then transformed into standardized scores (SS50), rescaling each measure to range from 0 to 50, with higher scores indicating greater levels of perceived stigma. The transformation was performed using the following formula: ${SS}_{\text{5}\text{0}}=\frac{{SS}_{raw}\times \text{5}\text{0}}{n\times \text{3}}$ where *n* represents the number of items in the scale, and 3 is the maximum possible score for each item [17].

The mean and standard deviation of SS50 were calculated for the study population. Participants were classified as experiencing perceived stigma if their SS50 score exceeded the sample mean, which was used as the cut-off point. The use of the sample mean as a threshold for categorizing perceived stigma has been applied in previous studies [17–19].

### Independent variables

Three groups of independent variables were collected to assess their associations with perceived stigma among people with pTB. The first group encompassed personal characteristics, including gender (male/female), age (55 and lower/ over 55), religion (no religion/ religious affiliation (Buddhism or Christianity)), education level (lower to secondary school/ high school and above), marital status (married/ other (single, widow…)), occupation (employed with income/ unemployed/ retired), place of residence (rural/ urban areas), and monthly income (≤ 5,000,000 VND/ over 5,000,000 VND; which equals to 189.6 USD). The second group’s comprised disease status included the presence of comorbid chronic conditions (yes/ no), history of pTB (yes/ no), and level of knowledge regarding pTB prevention (good/ not good).

The third group of independent variables, participants’ knowledge of pTB was assessed using a set of six questions adapted from the WHO questionnaires on pTB knowledge, attitudes, and practices [20]. The knowledge score was derived from six questions covering pTB symptoms, transmission, and prevention, with a total possible score ranging from 0 to 11 [16]. The cut-off point used to categorize knowledge levels was based on the mean knowledge score of the study sample. Specifically, a score of 7.04 was used as the threshold: participants with scores ≥7.04 were classified as having good knowledge, while those with scores <7.04 were classified as having poor knowledge.

#### Qualitative study themes.

Three major thematic domains were identified. First, individual-level factors included socio-demographic characteristics (age, gender, marital status, religion, education, occupation, and tuberculosis history), TB knowledge, and patients’ lived experiences and perceptions; these factors shaped the extent and manifestation of perceived stigma among TB patients.

Second, sociocultural and policy-related factors captured the broader context influencing patients’ perceived stigma. These included local beliefs about TB transmission and treatment, social barriers in relationships within communities and workplaces, stigma and prejudice from others, as well as the enabling and constraining effects of policies, social protection systems, and access to TB care and treatment services.

Third, healthcare worker attitudes and behaviors reflected patients’ experiences of interactions with healthcare providers, including perceived inappropriate communication or actions, which contributed to their sense of stigma.

The first two domains were explored with all three participant groups, including healthcare staff and pTB patients. The third domain was discussed exclusively with TB patients.

### Data collection tool, technique, and procedure

#### Quantitative study.

The study employed a structured quantitative questionnaire to collect data from pTB patients. The questionnaire included sections of personal characteristics, pTB knowledge, and VTSS to assess the pTB patients’ thoughts and feelings regarding their tuberculosis status.

Participant recruitment was conducted by a team of four investigators, including two staff members from the General Planning Department (one holding a Bachelor of Public Health and the principal investigator, LTVT) and two nurses from the Department of Tropical Diseases. Importantly, the nurses involved were not engaged in the direct clinical care of tuberculosis (TB) patients in the Department of Pulmonary Diseases.

All investigators received standardized training from the principal investigator prior to data collection, which covered participant identification, study procedures, and data collection methods to ensure consistency.

To minimize potential recruitment bias and reduce any perceived coercion, investigators did not wear healthcare uniforms during recruitment and introduced themselves solely as members of the research team affiliated with the hospital. Furthermore, as the recruiting nurses had no clinical responsibility for the TB patients approached, this helped to limit the risk of undue influence or perceived obligation to participate.

During the study period, trained data collectors visited inpatient wards to identify and invite eligible participants to take part in the study. Structured questionnaires were administered through face-to-face interviews. Upon completion of each interview, data collectors reviewed the questionnaires to ensure completeness and to address any missing or unclear responses.

At the end of each day, all completed questionnaires were further checked by the principal investigator for quality control before being submitted for data entry.

#### Qualitative study.

The study employed semi-structured in-depth interview (IDI) guides organized around predefined themes to facilitate open and flexible discussions with participants. A total of 13 IDIs were conducted, including one interview with the Head of the Department of Pulmonary Diseases, six with doctors and nurses from the Department of Pulmonary Diseases, and six with inpatients who were identified as experiencing stigma based on their responses to the quantitative questionnaire and were recruited prior to hospital discharge. Interviews with healthcare workers were conducted in the Department’s administrative office.

Data collection followed an iterative process, with concurrent preliminary review and coding of interview data. After approximately 11 interviews with 7 healthcare staff and 4 patients, no new themes or substantive insights were identified. Two additional interviews with patients were conducted to confirm this observation. As these final interviews did not yield new information or alter the existing coding framework, the research team determined that data saturation had been achieved, resulting in a final sample of 13 IDIs. All interviews were conducted by LTVT, the principal investigator, audio-recorded with participants’ consent, and transcribed verbatim for analysis.

Three semi-structured in-depth interview (IDI) guides were developed for three participant groups: pTB patients, the Head of the Department of Pulmonary Diseases, and healthcare staff. The guides were designed to comprehensively capture all predefined qualitative themes. Each IDI was conducted in Vietnamese and lasted approximately 25–30 minutes.

### Data analysis

Questionnaires were reviewed for completeness and consistency prior data entry into the Epidata 3.0 database. The software SPSS 20.0 was used to analyze quantitative data. Descriptive statistics were described in tables showing frequencies and proportions. Multivariable logistic regressions were performed using the Enter method, with a significance level p less than 0.05.

All IDIs were audio-recorded, transcribed verbatim in Vietnamese, and checked for accuracy against the original recordings. Data were analyzed using a thematic analysis approach. An initial coding framework was developed based on the predefined themes in the interview guides, and was iteratively refined to incorporate emerging themes from the data.

Two members of the research team independently coded a subset of transcripts to ensure consistency, and discrepancies were discussed and resolved through consensus. The finalized coding framework was then applied to all transcripts. Codes were organized into categories and overarching themes using Mindmap software to facilitate visualization and comparison across participants.

Qualitative findings were used to triangulate, contextualize, and enrich the quantitative results, providing a more comprehensive understanding of factors associated with TB-stigma.

### Ethical consideration

The study protocol and data collection procedures adhered to international ethical standards and local regulations. Ethical approval of the study was obtained from the Institutional Review Board for Scientific Research of the Hanoi University of Public Health, under Decision number 170/2024/YTCC-HD3 dated May 9, 2024, prior to the commencement of data collection. Additionally, the study received administrative support from the Management Board of the Hue Central Hospital.

Written informed consent was obtained from all participants prior to enrollment. Participants were informed about the study objectives, procedures, voluntary participation, and their right to withdraw at any time without consequences. All information collected from the study participants was kept confidential and used solely for research purposes. Patients with pTB who were identified experiencing stigma were advised to seek appropriate support services available at the hospital.

## Results

### Characteristics of the study participants

Among the 200 participants, more than two-thirds were male (71.5%), had no religion (73.0%), and were married (87.5%). Nearly half of the patients were under 55 years old. The majority had completed high school or attained higher levels of education (65.5%), were employed with income (54.5%), and resided in rural areas (67.5%) (Table 1).

**Table 1:** Associations between personal characteristics and stigma among pTB patients (N = 200): multivariable logistic regression.

Variable	Subgroup	Total		Stigma (yes)		Multivariable regression			
						p	Adjusted OR	95% CI	
		N	%	N	%			Lower	Upper
Total		200	100	**87**	**43.5**				
Sex	Male	143	71.5	61	42.7	0.993	1.00	0.49	2.06
	Female	57	28.5	26	45.6	1			
Age	≤ 55	94	47.0	38	40.4	0.057	0.45	0.20	1.02
	>55	106	53.0	49	46.2	1			
Religion	No religion	146	73.0	64	43.8	0.926	0.97	0.47	1.99
	Religious (Buddhism or Christianity)	54	27.0	23	42.6	1			
Education	Lower to secondary school	69	34.5	28	40.6	0.734	0.89	0.44	1.79
	High school and higher	131	65.5	59	45.0	1			
Marital status	Other (single, widow...)*	25	12.5	14	56.0	**0.048**	**2.93**	**1.01**	**8.47**
	Married	175	87.5	73	41.7	1			
Occupation	Currently working with income	109	54.5	47	43.1	0.398	1.44	0.62	3.36
	Unemployment	20	10.0	10	50.0	0.698	1.27	0.38	4.24
	Retirement	66	33.0	28	42.4	1			
Living location	Rural areas	135	67.5	59	43.7	0.294	1.45	0.72	2.93
	Urban areas	65	32.5	28	43.1	1			
Income	> 5,000,000* (equaled to USD 189.6)	89	44.5	45	50.6	**0.046**	**2.01**	**1.01**	**3.99**
	≤ 5,000,000	111	55.5	42	37.8	1			
Chronic disease	Chronic diseases	119	59.5	51	42.9	0.839	1.07	0.56	2.03
	No other diseases	81	40.5	36	44.4	1			
History of TB	Yes, ever	42	21.0	14	33.3	0.170	0.59	0.28	1.25
	No, never	158	79.0	73	46.2	1			
TB knowledge	Not good	59	29.5	25	42.4	0.498	0.79	0.39	1.57
	Good	141	70.5	62	44.0	1			

* *p < 0.05*.

### Prevalence of perceived stigma among experienced by pulmonary tuberculosis inpatients

Table 2 presents the distribution of responses from 200 pulmonary tuberculosis (TB) patients to selected items from the Van Rie Tuberculosis Stigma Scale. A substantial proportion of participants expressed selective disclosure of their TB status, with 87% (11.5% strongly agree, 75.5% agree) reporting that they chose carefully whom they shared their diagnosis with. Emotional burden was also witnessed, as 27.5% of respondents (3% strongly agree, 24.5% agree) reported feelings of guilt related to the burden of care placed on their family. Similarly, stigma-related avoidance behavior was evidenced by 90.5% of respondents (25.5% strongly agree, 65% agree) who reported keeping a distance from others to prevent transmission.

**Table 2:** Perceived stigma of hospitalized patients (N = 200).

No	VTSS items	Strongly Agree		Agree		Disagree		Strongly Disagree	
		n	%	n	%	n	%	n	%
1	I feel hurt how others react to knowing I have TB	15	7.5	50	25.0	81	40.5	54	27.0
2	I lose friends when I share with them that I have TB	5	2.5	49	24.5	105	52.5	41	20.5
3	I feel alone	11	5.5	47	23.5	106	53.0	36	18.0
4	I keep a distance from others to avoid spreading TB germs	51	25.5	130	65.0	14	7.0	5	2.5
5	I am afraid to tell those outside my family that I have TB	7	3.5	46	23.0	100	50.0	47	23.5
6	I am afraid of going to TB clinics because other people might see me there	6	3.0	17	8.5	123	61.5	54	27.0
7	I feel guilty because my family has the burden of caring for me	6	3.0	49	24.5	101	50.5	44	22.0
8	I choose carefully who I tell about having TB	23	11.5	151	75.5	16	8.0	10	5.0
9	I am afraid to tell my family that I have TB	6	3.0	23	11.5	117	58.5	54	27.0

Fear of social repercussions was another salient theme, with 11.5% of participants expressed discomfort with the idea of being seen at TB clinics (3% strongly agree and 8.5% agree), and 8.5% indicated fear in disclosing their condition to those outside the family (3.2% strongly agree and 5.2% agree). Emotional distress was evident among 29% of participants (5.5% strongly agree, 23.5% agree) who reported feeling alone.

Negative interpersonal outcomes were also reported. More than a quarter agreed (24.5%) or strongly agreed (2.5%) that they lost friends due to disclosing their TB status, while 32.5% of participants strongly agreed or agreed that they felt hurt by others’ reactions. In contrast, fewer participants (14.5%) reported being afraid to tell their family.

In-depth interviews revealed that many patients experienced feelings of being stigmatized from those around them, including their family members. As a result, they reported a loss of confidence and expressed anxiety in relation to their illness. Some patients indicated a desire to conceal their condition to avoid social judgment or chose to withdraw from social interactions altogether.

*“If someone’s scared of me, I just stay away from them. If they’re not, then it’s fine. A lot of my family members are actually scared of me.”* (IDI_04_Female patient).

*“Self-stigma feels like people think there’s something about me they don’t like because of my illness. I usually don’t tell people what I’m dealing with.”* (IDI_05_Male patient).

Due to the lack of psychological support and effective communication, patients experienced difficulties in expressing their feelings and discussing their illness. This presented a potential challenge in the processes of pTB diagnosis and treatment.

*“Honestly, I don’t really understand everything, but... I do know about the problem...but...I feel.., I feel like I get it but I can’t really explain it clearly. I feel like I know.”* (IDI_06_Male patient).

*“Sometimes when I ask them, they hide their illness... they are scared to talk about their symptoms.”* (IDI_04_Nurse).

Some patients chose to pay out-of-pocket for treatment at tertiary hospitals instead of utilizing services covered by universal healthcare insurance. The decision was driven by a lack of confidence in the capacity of health facilities in the health insurance program. These patients expressed a desire to be cured as quickly as possible.

*“I went to the hospital for an examination because I thought, if I go there, I’ll just pay a few hundred thousand and at least feel safe. But if I go to those smaller hospitals, I get worried about the results they might give me.”* (IDI_03_Female patient).

**Fig 1:**
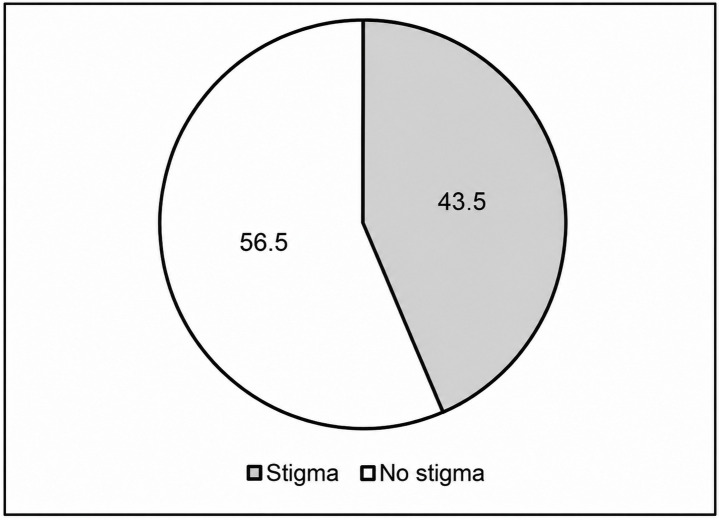
Prevalence of participants with perceived stigma measured by VTSS scale (n = 200).

The mean standardized total score (SS50) of the study sample was 21 (± [8]), with scores ranging from 7 to 50. The prevalence of patients with perceived stigma accounted for 43.5% of the total patients participating in the study (Fig 1).

### Factors associated with perceived stigma among pTB patients

#### Personal characteristics.

Table 1 presents the results of a multivariable logistic regression analysis examining the association between the presence of perceived stigma and several personal characteristics, health conditions and TB knowledge among study participants. The findings indicate that pTB patients who were not in a relationship had significantly higher odds of experiencing perceived stigma compared to those who were married (aOR = 2.93, 95%CI: 1.01 – 8.47, p = 0.048). Additionally, patients with a monthly income above 5 million VND were more likely to report perceived stigma compared to those with lower income levels (aOR = 2.01, 95% CI: 1.01 – 3.99, p = 0.046). Although a higher prevalence of perceived stigma was observed among older participants, those with higher education levels, those who were unemployed, and those who had never had pTB in the past, these differences were statistically insignificant.

#### Relationship with family and community.

Qualitative results indicate that negative relationships between patients with pTB and their families and communities had a detrimental impact on patients’ perceived stigma. The absence of family’s and relatives’ support, especially for older patients, contributed to their feeling of being abandoned. In addition, due to fear of transmission, some of their family members deliberately distanced themselves from the patients or neglected them. This situation exacerbated patients’ sense of isolation and inferiority.

*“My wife only comes with me when I ask her to, otherwise, she just stays home... because of housework.”* (IDI_02_Male patient).

*“Many family members are afraid of me.”* (IDI_04_Female patient).

*“There are people who even abandon the patients when the illness gets too severe, there are cases like that,”* (IDI_02_Doctor).

Beyond familial context, social stigma in the community also contributed to the isolation of pTB patients. These patients were frequently avoided upon exhibiting symptoms such as coughing, even prior to a confirmed diagnosis of pTB. Prevailing social prejudice regarding pTB was common and had direct impacts on patients’ self-confidence and social isolation, thereby developing and reinforcing patients’ stigmatization.

*“In society, social stigma against pulmonary tuberculosis is still really strong. When people hear someone has it, they tend to avoid them.”* (IDI_05_Nurse).

*“When people find out someone has tuberculosis or even just a persistent cough, they tend to avoid them because they are scared of getting infected.”* (IDI_03_Doctor).

*“Self-stigma is even worse than stigma from others... Patients start to feel like everyone’s watching them, judging them, or even afraid of them.”* (IDI_04_Nurse).

Community stigma also negatively affects patients’ ability to maintain employment, disrupts their participation in social activities, and fosters a sense of being a burden to their family.

*“More or less, everything is hindered... having to call in sick, asking for leave, etc., it takes up to a month.”* (IDI_04_Nurse).

#### The national TB program and other support for pTB patients.

Qualitative IDIs show that there were various limitations in the current TB prevention program, which affected the treatment process of pTB patients and increased their feelings of inferiority, anxiety and stigma. Although health insurance covered most of pTB treatment cost, limitations remained regarding medications for patients with co-morbidities. For pTB patients with co-morbidities such as hepatitis or kidney failure, adherence to the standard pTB treatment regimen would be challenging. These patients were unable to use the fixed-dose combination pill due to adverse side effects, necessitating the purchase of other drugs that might not be covered by health insurance. This situation increased patients’ helplessness and financial concern, thereby heightening their self-consciousness about their pTB condition.

*“Patients with so-called co-morbidities can’t use the mixed-dose combination pill in the National TB Prevention Program.”* (IDI_03_Doctor).

*“They have to switch their medicine and pay for it themselves because not everyone can get the medicine covered by the Health Insurance. This affects the patients, they get frustrated and don’t want to continue the treatment”.* (IDI_01_Doctor).

In addition, following the intensive treatment phase and discharge from the hospital, patients encountered difficulties in continuing treatment at health facilities at lower levels due to a complicated management process and lack of close monitoring from health professionals. Moreover, patients had insufficient understanding of, nor were they informed about the decentralized management of the National Tuberculosis Program, in which they would be transferred to health facilities at lower levels for follow-up care after completing intensive treatment at specialized TB hospitals. As a result, they might feel confused and exhausted when navigating administrative procedures across different levels of care, which disrupted their daily lives and work, and exacerbated their feeling of isolation and stigma.

*“Some patients go to their local area to get medicine, but they don’t understand the process... Then they encounter problems, so they come here to ask for help before going back to their locality”* (IDI_06_Doctor).

Prolonged treatment duration and limited access to periodical medical check-ups were also significant barriers for patients residing far from healthcare facilities or experiencing financial difficulties. These challenges often resulted in disease exacerbation, thereby creating a vicious cycle in which incomplete treatment worsened their conditions and reinforced their stigmatized conditions.

*“Many times, they don’t come to get their medicine because it’s hard to travel, so they skip it, sometimes letting the illness get worse.”* (IDI_04_Nurse).

#### Attitudes and behaviors of healthcare workers in the Department of Pulmonary Disease, Hue Central Hospital.

The behavior and attitudes of healthcare workers play an important role in reducing stigma among patients with pTB. In addition to providing treatment, the medical staff at Hue Central Hospital were willing to answer patients’ questions and created an open environment. This was helpful in reducing patients’ feelings of inferiority and isolation during treatment. Most interviewed patients expressed satisfaction and positive feelings regarding the quality of care at Hue Central Hospital. They reported feeling more comfortable and confident during the treatment process.

*“Yes, in general, the nurses here are quite good and friendly,”* (IDI_02_Male patient).

*“People say that doctors are like mothers, they always take care of patients like they are their own family members.”* (IDI_01_Male patient).

*“I think the doctors and nurses here are really dedicated. Whenever I ask them something, they are always very helpful.”* (IDI_03_Female patient).

## Discussion

### Experience of perceived stigma among hospitalized pTB patients

Our study reveals a substantial psychosocial burden among pulmonary TB inpatients, with 43.5% experiencing perceived stigma. This finding represents a critical threshold of internalized and experienced distress. While this prevalence is lower than community-based findings in Cambodia (>50%) [21] and Indonesia (45.9%) [22], the significance lies in the qualitative insights of the Vietnamese context. We argue that the supportive environment at Hue Central Hospital mitigates visible acts of discrimination, yet perceived stigma remains profoundly high.

Specifically, the 27.5% prevalence of guilt over being a family burden suggests that patients do not merely perceive external prejudice but deeply internalize negative societal narratives. This internal struggle is closely linked to the cultural expectation of protecting family reputation. In Vietnam, the family is often viewed as a single moral unit; thus, a TB diagnosis is not just an individual illness but a collective social burden. This explains the 8.5% rate of selective disclosure - a protective mechanism of secrecy that aligns with the anticipated stigma findings by Redwood et al. (2022) [9]. According to this perspective, patients often manage a complex socially responsible identity, isolating themselves to preserve their family’s standing and protect their social circle from courtesy stigma.

Furthermore, the 24.5% rate of social network loss (enacted stigma) and 11.5% fear of being seen at clinics (anticipated stigma) further illustrate the multidimensional nature of perceived stigma. These figures demonstrate that clinical cure does not automatically translate to social reintegration. As theorized by Courtwright and Turner (2010) [23], the physical infrastructure of TB care - centralized and highly recognizable - can unintentionally reinforce perceived structural stigma. In a national-level hospital, the high visibility of seeking care can compromise a patient’s privacy. For many, the pursuit of life-saving treatment entails a constant tension between clinical recovery and the potential loss of social prestige. This visibility-risk is a critical barrier identified in other high-burden settings like Ethiopia [24], where the fear of accidental disclosure at health facilities often drives anticipated stigma, leading to delayed care-seeking and psychological withdrawal.

### Factors associated with perceived stigma among pTB inpatients

A notable finding of our study is the socioeconomic contradiction where higher income was significantly linked to increased perceived stigma. This finding contrasts sharply with the global trend where poverty is the primary driver of stigma due to limited resources and education [24]. By integrating our qualitative results, we explain this through the framework of social status vulnerability. For higher-income patients in Vietnam, the professional and social consequences of a TB diagnosis are perceived as much more severe. Unlike patients with fewer resources who may prioritize immediate survival, those with higher social capital face a more profound threat to their professional standing and future marital prospects [8].

The association between unmarried/widowed status and higher perceived stigma further underscores the family’s role as a protective support mechanism. Consistent with Adejumo et al. (2025) [25] and Chen et al. (2021) [26], our data suggests that stable familial support provides a psychosocial defense that reduces anticipated stigma and improves quality of life for patients. Conversely, unmarried, or socially isolated patients lack this intimate support system, making them more vulnerable to the internalization of community prejudice.

These individual vulnerabilities are significantly influenced by the broader institutional environment. International literature, for example Bodur and Çil (2025) [11], often highlights adversarial healthcare interactions, where negative attitudes from staff exacerbate patient’s perceived stigma. However, our study offers a critical counter-perspective. The positive experiences and empathetic, personalized care reported by our participants suggest that high-quality, patient-centered interactions at the tertiary level can actively deconstruct perceived institutional stigma. This clinical partnership is a powerful tool for retention, yet it is often undermined by structural constraints.

As evidenced by PATH (2023) [4] and Forse et al. (2024) [5], fragmented decentralized care and complex health insurance policies for patients with comorbidities create systemic structural challenges that fuel anticipated stigma. When patients struggle to access services or face unexpected financial costs, their sense of vulnerability and perceived stigma increases. Therefore, as proposed by Nuttall et al. (2022) [12], reducing perceived stigma must be a multi-level effort. It requires not only training healthcare workers in stigma-sensitive communication but also optimizing the structural pathways to care. Addressing TB stigma in Vietnam thus necessitates a dual strategy, including preserving the supportive familial and clinical environments and reforming the systemic financial barriers that compromise patient dignity.

### Limitations of the study

Several epidemiological and methodological limitations should be noted in this study. First, selection bias (specifically Berkson’s bias) may exist, as our sample consisted of hospitalized patients at a single tertiary hospital who may have different profiles than TB patients in the community, potentially affecting the generalizability of the observed perceived stigma levels. Second, the study is subject to information bias, particularly interviewer bias, as data collection by hospital staff may have influenced responses due to perceived authority or the cultural emphasis on family reputation, leading to a possible underreporting of internalized or anticipated stigma. Third, while we utilized qualitative themes to contextualize and explain the quantitative associations - such as the link between socioeconomic status and perceived stigma - the depth of this mixed-methods integration could be further enhanced. Future research might benefit from a more iterative design where qualitative findings are used to prospectively refine quantitative instruments. Additionally, our regression model faces residual confounding due to the omission of variables like illness duration and treatment phase, which may independently influence perceived stigma. The absence of perspectives from patients’ relatives also limits our understanding of how familial dynamics shape perceived stigma. Furthermore, we acknowledge that the use of a sample-dependent cut-off to define the presence of perceived stigma may influence the estimated prevalence. This approach, while internally consistent for our study participants, may limit direct comparability with other research employing alternative thresholds or different scoring methodologies. Consequently, the reported prevalence should be interpreted within the specific context of this study’s population and the measurement parameters of the Van Rie TB Stigma Scale. Finally, the cross-sectional design prevents establishing temporal precedence, raising the possibility of reverse causality in the associations between social factors and perceived stigma.

## Conclusion and recommendations

Our study showed that perceived stigma was a major psychosocial problem of pTB patients in Vietnam, driven by a complex association of individual vulnerabilities such as lack of family/spousal support, persistent community prejudice, and critical structural deficiencies in healthcare financing and coordination. These findings highlight that perceived stigma is not merely an individual psychological concern but a systemic issue that necessitates integrated interventions, ranging from enhanced social support mechanisms to structural reforms in the national TB control program.

It is suggested by our study that future programs should deploy community-based interventions and utilize independent (non-clinical) social workers for counseling to ensure a more representative reach and minimize social desirability bias during data collection. Longitudinal studies are essential to adjust for illness duration and treatment phases, thereby establishing clear temporal directions and controlling for residual confounding. Stigma-reduction strategies must shift from patient-only models to family-centered approaches to address the domestic isolation currently uncaptured by hospital-centric care. Finally, to reduce the systemic barriers and disclosure risks found in this inpatient population, the National TB programs should facilitate referral pathways and implement institutional-wide stigma training for all healthcare levels.

## Supporting information

S1 Data Study dataset. (SAV)

## References

[1] World Health Organization. Tuberculosis - fact sheet. Available from: https://www.who.int/news-room/fact-sheets/detail/tuberculosis. 2025. Accessed 2025 November 20

[2] World Health Organization. Global tuberculosis report 2025. Geneva: WHO; 2025.

[3] Çil B, Bodur MS, Kabak M. Diagnostic shifts in extrapulmonary tuberculosis during COVID-19: evidence of vulnerability among migrants in a border province. BMC Infect Dis. 2026;26(1):646. doi: 10.1186/s12879-026-12909-3 41715019 PMC13023141

[4] PATH. Barriers to access and use of public TB diagnostic services in Vietnam. Available from: https://media.path.org/documents/CP_vietnam_barriers_tb_rpt.pdf. 2023. Accessed 2026 August 1

[5] Forse R, Yoshino CA, Nguyen TT, Phan THY, Vo LNQ, Codlin AJ, et al. Towards universal health coverage in Vietnam: a mixed-method case study of enrolling people with tuberculosis into social health insurance. Health Res Policy Syst. 2024;22(1):40. doi: 10.1186/s12961-024-01132-8 38566224 PMC10985876

[6] Scambler G. Health-related stigma. Sociol Health Illn. 2009;31(3):441–55. doi: 10.1111/j.1467-9566.2009.01161.x 19366430

[7] Weiss MG, Ramakrishna J, Somma D. Health-related stigma: rethinking concepts and interventions. Psychol Health Med. 2006;11(3):277–87. doi: 10.1080/13548500600595053 17130065

[8] Le Ngoc H, Le Minh G, Nguyen Binh H, Dinh Van L. Knowledge, attitudes, practices, and the post-cure stigma paradox: determinants of Van Rie stigma scores among MDR-TB patients in Vietnam. Available from: https://www.preprints.org/manuscript/202604.1134/v1. 2026. Accessed 2026 May 14

[9] Redwood L, Fox GJ, Nguyen TA, Bernarys S, Mason P, Vu VA, et al. Good citizens, perfect patients, and family reputation: stigma and prolonged isolation in people with drug-resistant tuberculosis in Vietnam. PLOS Glob Public Health. 2022;2(6):e0000681. doi: 10.1371/journal.pgph.0000681 36962771 PMC10021913

[10] Chaychoowong K, Watson R, Barrett DI. Perceptions of stigma among pulmonary tuberculosis patients in Thailand, and the links to delays in accessing healthcare. J Infect Prev. 2023;24(2):77–82. doi: 10.1177/17571774231152720 36815061 PMC9940242

[11] Bodur MS, Çil B. A Research on healthcare professionals’ stigma towards tuberculosis patients. Thorac Res Pract. 2025;26(3):88–96. doi: 10.4274/ThoracResPract.2024.24079 39930730 PMC12047200

[12] Nuttall C, Fuady A, Nuttall H, Dixit K, Mansyur M, Wingfield T. Interventions pathways to reduce tuberculosis-related stigma: a literature review and conceptual framework. Infect Dis Poverty. 2022;11(1):101. doi: 10.1186/s40249-022-01021-8 36138434 PMC9502609

[13] Redwood L, Mitchell EMH, Nguyen TA, Viney K, Duong L, Phạm HT, et al. Adaptation and validation of the Van Rie tuberculosis stigma scale in Vietnam. Int J Infect Dis. 2022;114:97–104. doi: 10.1016/j.ijid.2021.10.040 34715359

[14] Hue Central Hospital, Department of Pulmonology. Summary of professional activities in 2024 and directions for activities in 2025; 2024.

[15] Hue Central Hospital. General introduction. Available from: https://bvtwhue.com.vn/Danhmuc/Baiviet/?lang=eng&ID=20042. Accessed 2026 January 5

[16] Teo AKJ, Prem K, Tuot S, Ork C, Eng S, Pande T, et al. Mobilising community networks for early identification of tuberculosis and treatment initiation in Cambodia: an evaluation of a seed-and-recruit model. ERJ Open Res. 2020;6(2):00368–2019. doi: 10.1183/23120541.00368-2019 32391397 PMC7196668

[17] Van Rie A, Sengupta S, Pungrassami P, Balthip Q, Choonuan S, Kasetjaroen Y, et al. Measuring stigma associated with tuberculosis and HIV/AIDS in southern Thailand: exploratory and confirmatory factor analyses of two new scales. Trop Med Int Health. 2008;13(1):21–30. doi: 10.1111/j.1365-3156.2007.01971.x 18290998

[18] Abebe G, Deribew A, Apers L, Woldemichael K, Shiffa J, Tesfaye M, et al. Knowledge, health seeking behavior and perceived stigma towards tuberculosis among tuberculosis suspects in a rural community in southwest Ethiopia. PLoS One. 2010;5(10):e13339. doi: 10.1371/journal.pone.0013339 20948963 PMC2952624

[19] Duko B, Bedaso A, Ayano G, Yohannis Z. Perceived stigma and associated factors among patient with tuberculosis, Wolaita Sodo, Ethiopia: cross-sectional study. Tuberc Res Treat. 2019;2019:5917537. doi: 10.1155/2019/5917537 31186957 PMC6521372

[20] World Health Organization, Stop TB Partnership. Advocacy, communication and social mobilization for TB control: a guide to developing knowledge, attitude and practice surveys. 2008. Available from: https://iris.who.int/handle/10665/43790

[21] Teo AKJ, Tan RKJ, Smyth C, Soltan V, Eng S, Ork C, et al. Characterizing and measuring tuberculosis stigma in the community: a mixed-methods study in Cambodia. Open Forum Infect Dis. 2020;7(10):ofaa422. doi: 10.1093/ofid/ofaa422 33134412 PMC7585330

[22] Megawati D, Ainul Yaqin Y, Yunita R. The relationship between self-stigma and subjective well-being in tuberculosis patients. HTechJ. 2024;2(5):516–25. doi: 10.53713/htechj.v2i5.267

[23] Courtwright A, Turner AN. Tuberculosis and stigmatization: pathways and interventions. Public Health Rep. 2010;125 Suppl 4(Suppl 4):34–42. doi: 10.1177/00333549101250S407 20626191 PMC2882973

[24] Datiko DG, Jerene D, Suarez P. Stigma matters in ending tuberculosis: nationwide survey of stigma in Ethiopia. BMC Public Health. 2020;20(1):190. doi: 10.1186/s12889-019-7915-6 32028914 PMC7006204

[25] Adejumo OA, Jinabhai C, Daniel O, Haffejee F. The effects of stigma and social support on the health-related quality of life of people with drug resistance tuberculosis in Lagos, Nigeria. Qual Life Res. 2025;34(5):1305–16. doi: 10.1007/s11136-025-03902-5 39964366 PMC12064618

[26] Chen X, Du L, Wu R, Xu J, Ji H, Zhang Y, et al. Tuberculosis-related stigma and its determinants in Dalian, Northeast China: a cross-sectional study. BMC Public Health. 2021;21(1):6. doi: 10.1186/s12889-020-10055-2 33397334 PMC7780403

