# Supplementary material for: Perceived stigma among hospitalized pulmonary tuberculosis patients in Hue Central Hospital: A 2024 mixed method study in Vietnam
Source: PLOS Glob Public Health. 2026 Jul 13;6(7):e0006752. doi: 10.1371/journal.pgph.0006752 (PMC13362144; doi:10.1371/journal.pgph.0006752)
Supplement: Attachment — Submitted filename: Comments- Plos Global Public Health.docx [file pgph.0006752.s002.docx]

Thank you for the opportunity to review the paper titled **‘Stigma of hospitalized pulmonary tuberculosis patients in Hue Central Hospital: a 2024 mixed method study in Vietnam’**. Some of my suggestions to improve its rigor are provided below.

**Aim of the study:** The aim has been mentioned as to **estimate the prevalence** of stigma and identify factors in the Abstract, while the aim mentioned in the Introduction is to **describe the status** of stigma and associated factors. Given the mixed methods design, this is acceptable, but make it consistent in both the abstract and the introduction.

**Study design, time, and location**: Why Hue Central Hospital? Justify the selection of this study setting and briefly describe its location and characteristics, such as facilities and treatments available for TB patients, so that readers understand the context.

**Study participants:** Who made the participant recruitment, and what was the procedure? Was there any involvement by the hospital staff in recruiting patients for the study, if so, how did you avoid any potential bias?

**Sample size and selection of participants:** You have conducted 13 in-depth interviews. Did you conduct interviews until data saturation was reached? If so, how did you decide the point of saturation?

**Qualitative study themes**- You have a sub-heading: ‘qualitative study themes’ which is not clear. No themes are mentioned. You can remove the term ‘themes’ from here.

**Data collection tool, technique, and procedure**: It seems your questionnaire is interviewer-administered; please clarify.

**Qualitative study**: You have mentioned that ‘a set of qualitative in-depth interviews (IDI) guides’ were used, which is not clear. How many interview guides were developed and for whom? How long was an interview? What was the language in which you conducted the interviews?

**Data analysis:** Please elaborate on how you performed the qualitative data analysis. In one place, you had mentioned that the interview guide was developed based on some pre-determined themes. Then, what were they, and how did you analyze the data?

**Results:** You have a theme mentioned as ‘Attitudes and behaviors of healthcare workers in the Pneumonia Disease Department, Hue Central Hospital’. Do you have any quotes from healthcare workers to support the findings?
