# Supplementary material for: Perceived stigma among hospitalized pulmonary tuberculosis patients in Hue Central Hospital: A 2024 mixed method study in Vietnam
Source: PLOS Glob Public Health. 2026 Jul 13;6(7):e0006752. doi: 10.1371/journal.pgph.0006752 (PMC13362144; doi:10.1371/journal.pgph.0006752)
Supplement: Attachment — Submitted filename: 4. Response_to_reviewers.docx [file pgph.0006752.s003.docx]

**Manuscript title: Perceived stigma among hospitalized pulmonary tuberculosis patients in Hue Central Hospital: a 2024 mixed method study in Vietnam**

**Manuscript Number: PGPH-D-26-00084**

| **Reviewer Remarks** | **Author comments** |
| --- | --- |
| **1. From editor** | |
| We note that you have indicated that there are restrictions to data sharing for this study. For studies involving human research participant data or other sensitive data, we encourage authors to share de-identified or anonymized data. However, when data cannot be publicly shared for ethical reasons, we allow authors to make their data sets available upon request. | We thank the Editor for this guidance. We are committed to data transparency and have now provided the de-identified and anonymized dataset as a supporting information file alongside this revised manuscript. We confirm that all personal identifiers have been removed to ensure participant confidentiality in accordance with ethical standards |
| Before we proceed with your manuscript, please address the following prompts:  a) If there are ethical or legal restrictions on sharing a de-identified data set, please explain them in detail (e.g., data contain potentially identifying or sensitive patient information, data are owned by a third-party organization, etc.) and who has imposed them (e.g., a Research Ethics Committee or Institutional Review Board, etc.). Please also provide contact information for a data access committee, ethics committee, or other institutional body to which data requests may be sent.  b) If there are no restrictions, please upload the minimal anonymized data set necessary to replicate your study findings to a stable, public repository and provide us with the relevant URLs, DOIs, or accession numbers. | We appreciate the Editor’s clarification regarding the data availability policy.  We confirm that there are no ethical or legal restrictions that prevent the sharing of an anonymized dataset. As mentioned above, we have now prepared the anonymized dataset required to replicate our study findings, ensuring that all sensitive and potentially identifying patient information has been removed.  This dataset has been uploaded as a supporting information file alongside the revised manuscript. We believe this fulfills the journal's requirements for data transparency while maintaining strict participant confidentiality as approved by our Institutional Review Board. |
| Please provide separate main figure files in .tif or .eps format only and ensure that all files are under our size limit of 10MB. | We have followed the journal's technical requirements regarding figure preparation. All main figures have been converted into .tif forma**t** (or .eps) with a minimum resolution of 300 DPI to ensure high quality. We also confirm that each separate figure file is under the 10MB size limit. These files have been uploaded individually through the submission system |
| The ethics approval documents should not be published with the paper and are for internal information only. Please change the file type to 'Other' to ensure that they are not published. | We have noted the Editor’s instruction regarding the ethics approval documents. Accordingly, we have updated the file type for all ethical clearance documents to 'Other' in the submission system. This ensures that these documents remain available for internal editorial review only and will not be included in the final publication. |
| **2. Reviewer 1** | |
| The introduction would benefit from a stronger integration with recent epidemiological literature on tuberculosis vulnerability and healthcare access. Tuberculosis-related stigma does not occur in isolation but is closely linked to structural determinants such as diagnostic practices, health system capacity, and the social context of vulnerable populations. Recent studies have highlighted how diagnostic inequalities and structural barriers may influence both the clinical course of tuberculosis and the psychosocial experiences of affected patients. In this regard, the authors may consider integrating recent studies examining tuberculosis epidemiology, diagnostic access, and healthcare utilization, including work demonstrating diagnostic shifts and testing gaps in tuberculosis care. For example, the study by Çil et al. examining diagnostic shifts in extrapulmonary tuberculosis during the COVID-19 period highlights how health system disruptions and population vulnerabilities can influence tuberculosis diagnosis and care pathways (Çil B, Bodur MS, Kabak M. Diagnostic shifts in extrapulmonary tuberculosis during COVID-19: evidence of vulnerability among migrants in a border province. BMC Infectious Diseases. 2026). Similarly, evidence demonstrating underutilization of bacteriological testing in extrapulmonary tuberculosis suggests that diagnostic practices remain inconsistent across healthcare settings and may contribute to delayed diagnosis and social vulnerability among tuberculosis patients (Çil B, Bodur MS, Kabak M. Underutilization of Bacteriological Testing in Extrapulmonary Tuberculosis: A Retrospective Single-center Study from Mardin, Türkiye. Thoracic Research and Practice. 2026). Incorporating such literature would strengthen the conceptual background and provide a broader perspective on structural determinants influencing tuberculosis care. | We sincerely thank the reviewer for this constructive suggestion, which has significantly enhanced the conceptual depth of our Introduction. We agree that TB-related stigma must be examined within the broader context of structural determinants and health system capacity.  Accordingly, we have revised and rewritten the Introduction to integrate recent epidemiological literature and structural perspectives:   - Integration of diagnostic vulnerability: As suggested by the reviewer, we have incorporated the work of Çil et al. (2026) to demonstrate how health system disruptions, diagnostic shifts exacerbate the vulnerability of TB patients (references 3). We now argue that these structural inconsistencies not only affect the clinical course but also shape the psychosocial experiences of patients by facilitating delayed diagnosis and social marginalization. - Structural barriers in Vietnam: We have added recent evidence regarding healthcare utilization in Vietnam, citing PATH (2023) on barriers to public diagnostic services and Forse et al. (2024) on the challenges of enrolling TB patients into social health insurance (references 4 and 5, respectively). These additions highlight how geographical, administrative, and economic factors act as structural determinants of both care access and stigma. - Broadened perspective: By linking stigma to diagnostic practices and system capacity, the Introduction now provides a more comprehensive rationale for why a national tertiary institution like Hue Central Hospital is a critical setting for this study.   We believe these revisions provide a stronger bridge between TB epidemiology and the psychosocial impact of the disease, as requested by the reviewer. |
| The manuscript would benefit from clearer conceptual differentiation between different forms of stigma. The study frequently refers to “stigma” as a single construct; however, tuberculosis-related stigma can manifest in multiple forms including perceived stigma, internalized stigma, enacted stigma, and institutional stigma. Several of the behaviors reported in the study appear to reflect internalized or anticipated stigma rather than direct discriminatory experiences. For example, the finding that more than 90% of participants reported intentionally keeping distance from others may partly reflect infection-control awareness rather than stigma alone. Distinguishing these dimensions would allow a more nuanced interpretation of the findings and would align the study with current conceptual frameworks used in tuberculosis stigma research. | We thank the reviewer for this insightful suggestion. We have revised, rewritten the introduction and revised the manuscript to provide a clearer conceptual differentiation between the various dimensions of stigma, as requested:   - Theoretical framework: We have revised and rewritten the Introduction to explicitly define and distinguish between enacted stigma (direct discrimination), anticipated stigma (fear of rejection), and internalized stigma (self-stigma/shame), drawing on the frameworks of Scambler (2009) and Weiss et al. (2006) (references 6 and 7, respectively). - Nuanced interpretation of findings: We have addressed the reviewer’s point regarding patients 'keeping distance.' In the revised Discussion, we now acknowledge that this behavior (reported by over 90% of participants) is a complex intersection of legitimate infection-control awareness and anticipated stigma. We explain that while this reflects social responsibility, it is often compounded by a fear of social 'status loss' inherent in the Vietnamese cultural context. - Structural and individual factors: We have also elaborated on how individual characteristics and structural factors (such as the high visibility of a national-tier hospital) specifically trigger different forms of stigma - particularly anticipated stigma among patients from higher socioeconomic backgrounds.   These changes ensure that stigma is no longer treated as a single construct but as a nuanced phenomenon, aligning the study with current conceptual frameworks in TB research. |
| The classification approach used to define stigma prevalence requires clarification. The authors define stigma as present when the raw score exceeds the standardized summary score (SSraw > SS50). This method differs from commonly used scoring strategies in studies utilizing the Van Rie Tuberculosis Stigma Scale. Additional justification for this threshold should therefore be provided, including references supporting this approach and a discussion of how this classification may influence the reported prevalence of stigma in the study population. Without clarification, the estimate that 43.5% of hospitalized pulmonary tuberculosis patients experience stigma may be difficult to interpret in comparison with other studies. | Thank you for this important comment. We agree that the previously described classification approach may cause confusion and limit comparability with existing studies using the Van Rie Tuberculosis Stigma Scale.  We have revised the manuscript to clarify that stigma was not defined based on a comparison between SSraw and SS50. Instead, the cut-off score for stigma was determined using the sample mean of the standardized score (SS50). Specifically, participants with SS50 scores above the sample mean were classified as experiencing stigma.  This approach has now been clearly described in the Methods section. We also acknowledge in the Discussion that using a sample-dependent cut-off may influence the estimated prevalence and limit direct comparability with studies employing alternative thresholds. |
| The study reports that higher-income patients experienced higher levels of stigma compared with lower-income patients (adjusted OR = 2.01). This finding contradicts much of the existing literature, where stigma is more commonly associated with poverty, limited access to healthcare, and lower socioeconomic status. The discussion should therefore provide a deeper interpretation of this result and consider potential explanations such as sampling bias due to the hospital-based study design, contextual social dynamics, or measurement limitations within the stigma scale. The qualitative findings provide valuable insights into structural barriers affecting tuberculosis patients, including fragmented referral pathways, difficulties navigating decentralized treatment systems, and financial challenges related to treatment of comorbidities. These structural barriers appear to play a significant role in shaping patients’ psychosocial experiences and may contribute to both treatment delays and increased stigma. Expanding the discussion of health system factors would strengthen the manuscript and align the findings with broader research examining healthcare access and tuberculosis vulnerability. | We sincerely thank the reviewer for this insightful comment. We have significantly expanded the Discussion (Paragraph 4) to interpret this socioeconomic contradiction through multiple lenses:   - Contextual social dynamics**:** We now explain this through the framework of social status vulnerability. Drawing on qualitative insights and recent literature (Le Ngoc Huy et al., 2026; Redwood et al., 2022), we argue that for higher-income patients in Vietnam, the perceived professional and social consequences of a TB diagnosis are more severe, as it threatens their social prestige and professional standing (references 8 and 9, respectively). - Sampling bias and design limitations: We have added a dedicated section in the Limitations to acknowledge that our hospital-based design (tertiary level) may attract a specific patient profile, potentially over-representing those with higher status who fear accidental disclosure in smaller community settings (Berkson’s bias). - Measurement context: We clarified that the VTSS scale may capture higher "anticipated stigma" among wealthy patients who have more social capital to lose, contrasted with poverty-driven stigma models seen in countries like Ethiopia (Datiko et al., 2020) (references 24).   We agree that structural barriers are fundamental to the patient's psychosocial experience. We have integrated a comprehensive discussion on health system factors (Paragraph 6 of the Discussion):   - Systemic navigational challenges: Using the framework proposed by Nuttall et al. (2022) and reports by PATH (2023), we now discuss how fragmented care pathways and complex insurance policies for comorbidities create a "navigation burden" that exacerbates patient anxiety and vulnerability (references 12 and 4, respectively). - Universal health coverage gaps: We have cited Forse et al. (2024) to highlight how financial constraints and decentralized system inefficiencies contribute to treatment difficulties, which in turn fuels the sense of stigma and isolation (references 5). - Clinical-structural synergy: We emphasize that while individual clinical care (at Hue Central Hospital) is perceived positively, these systemic barriers remain significant "upstream" factors that need reform to improve care engagement. |
| The manuscript would benefit from engagement with literature examining stigma among healthcare professionals themselves. Evidence suggests that healthcare workers’ attitudes and perceptions toward tuberculosis patients may influence patient experiences and treatment engagement. For instance, research exploring stigma among healthcare professionals has demonstrated that institutional attitudes can play a critical role in shaping patient experiences and healthcare-seeking behavior (Bodur MS, Çil B. A Research on Healthcare Professionals’ Stigma Towards Tuberculosis Patients. Thoracic Research and Practice. 2025). Integrating such literature would enrich the discussion and highlight the multidimensional nature of tuberculosis-related stigma within healthcare systems. | Thank you for your valuable comment and suggestion of appropriate references. We entirely agree that the attitudes of healthcare professionals are a critical dimension of institutional stigma. As suggested, we have enriched the Discussion by:   - Integrating healthcare professional stigma literature: We have cited Bodur and Çil (2025) to acknowledge that negative attitudes among healthcare workers often exacerbate patient distress and deter healthcare-seeking behavior globally (references 11). - Providing a countervailing perspective: We contrasted these international findings with our qualitative data, which revealed that the empathetic, personalized care at Hue Central Hospital acted as a protective factor, helping to deconstruct institutional stigma. - Highlighting multidimensionality: By including this literature, we have strengthened the argument that reducing TB stigma requires a dual approach: addressing both structural system barriers and the quality of patient-provider interactions. |
| **3. Reviewer 2** | |
| Aim of the study: The aim has been mentioned as to estimate the prevalence of stigma and identify factors in the Abstract, while the aim mentioned in the Introduction is to describe the status of stigma and associated factors. Given the mixed methods design, this is acceptable, but make it consistent in both the abstract and the introduction. | We thank the reviewer for pointing out the inconsistency in the study aims. We have now aligned the objectives in both the Abstract and the Introduction.  The revised aim is “to assess the prevalence of stigma among hospitalized pTB patients and identify associated factors in Hue Central Hospital in 2024 through a mixed-methods approach”.  This phrasing consistently reflects both the quantitative and qualitative components of our study design |
| Study design, time, and location: Why Hue Central Hospital? Justify the selection of this study setting and briefly describe its location and characteristics, such as facilities and treatments available for TB patients, so that readers understand the context. | We thank the reviewer for the suggestion to further justify the study setting. We have now added a detailed description of Hue Central Hospital in the Methods section.  The selection of Hue Central Hospital was strategic for several reasons: (1) Its status as a leading tertiary-level national hospital allows for the study of stigma in a high-visibility clinical setting in the Central of Vietnam; (2) It serves a vast geographical catchment area (Central and Highland Vietnam), providing a diverse cohort of patients; and (3) Its comprehensive facilities (specialized isolation, advanced diagnostics) represent the highest standard of TB care in Vietnam. This context is crucial for understanding the 'status vulnerability' identified in our study, as patients at this level often face higher anticipated stigma due to the clinical visibility and the perceived severity of their condition when treated at a national-tier hospital. |
| Study participants: Who made the participant recruitment, and what was the procedure? Was there any involvement by the hospital staff in recruiting patients for the study, if so, how did you avoid any potential bias? | We thank the reviewer for this comment. The quantitative data collection procedure was presented in the Data collection tool, technique, and procedure/Quantitative study section. However, we revised such part as following to clarify the recruitment process and method to avoid recruitment bias:  “Participant recruitment was conducted by a team of four investigators, including two staff members from the General Planning Department (one holding a Bachelor of Public Health and the principal investigator, LTVT) and two nurses from the Department of Tropical Diseases. Importantly, the nurses involved were not engaged in the direct clinical care of tuberculosis (TB) patients in the Department of Pulmonary Diseases.  All investigators received standardized training from the principal investigator prior to data collection, which covered participant identification, study procedures, and data collection methods to ensure consistency.  To minimize potential recruitment bias and reduce any perceived coercion, investigators did not wear healthcare uniforms during recruitment and introduced themselves solely as members of the research team affiliated with the hospital. Furthermore, as the recruiting nurses had no clinical responsibility for the TB patients approached, this helped to limit the risk of undue influence or perceived obligation to participate.” |
| Sample size and selection of participants: You have conducted 13 in-depth interviews. Did you conduct interviews until data saturation was reached? If so, how did you decide the point of saturation? | We thank the reviewer for this comment.  Yes, interviews were conducted until data saturation was achieved. Data saturation was defined as the point at which no new themes, codes, or insights emerged from successive interviews. Throughout the data collection process, the research team conducted ongoing, iterative reviews of the interview data, including preliminary coding and discussion after each set of interviews.  We revised this part as following to clarify the data collection procedure of the qualitative component:  “The study employed semi-structured in-depth interview (IDI) guides organized around predefined themes to facilitate open and flexible discussions with participants. A total of 13 IDIs were conducted, including one interview with the Department Head, six with doctors and nurses from the Department of Pulmonary Diseases, and six with inpatients who were identified as experiencing stigma based on their responses to the quantitative questionnaire and were recruited prior to hospital discharge. Interviews with healthcare workers were conducted in the Department’s administrative office.  Data collection followed an iterative process, with concurrent preliminary review and coding of interview data. After approximately 11 interviews with 7 healthcare staff and 4 patients, no new themes or substantive insights were identified. Two additional interviews with patients were conducted to confirm this observation. As these final interviews did not yield new information or alter the existing coding framework, the research team determined that data saturation had been achieved, resulting in a final sample of 13 IDIs.  All interviews were conducted by LTVT, the principal investigator, audio-recorded with participants’ consent, and transcribed verbatim for analysis.” |
| Qualitative study themes- You have a sub-heading: ‘qualitative study themes’ which is not clear. No themes are mentioned. You can remove the term ‘themes’ from here. | We thank the reviewer for this comment.  We revised this part in the Qualitative themes section as following to clarify the themes of the qualitative study:  “Three major thematic domains were identified. First, individual-level factors included socio-demographic characteristics (age, gender, marital status, religion, education, occupation, and tuberculosis history), TB knowledge, and patients’ lived experiences and perceptions; these factors shaped the extent and manifestation of perceived stigma among TB patients.  Second, sociocultural and policy-related factors captured the broader context influencing patients’ stigma. These included local beliefs about TB transmission and treatment, social barriers in relationships within communities and workplaces, stigma and prejudice from others, as well as the enabling and constraining effects of policies, social protection systems, and access to TB care and treatment services.  Third, healthcare worker attitudes and behaviors reflected patients’ experiences of interactions with healthcare providers, including perceived inappropriate communication or actions, which contributed to their sense of stigma.  The first two domains were explored with all three participant groups, including healthcare staff and TB patients. The third domain was discussed exclusively with TB patients” |
| Data collection tool, technique, and procedure: It seems your questionnaire is interviewer-administered; please clarify. | We make it clear that data were collected via face-to-face interview. |
| Qualitative study: You have mentioned that ‘a set of qualitative in-depth interviews (IDI) guides’ were used, which is not clear. How many interview guides were developed and for whom? How long was an interview? What was the language in which you conducted the interviews? | We added this information in the data collection section.  “Three semi-structured in-depth interview (IDI) guides were developed for three participant groups: TB patients, the Head of the Department of Pulmonary Diseases, and healthcare staff. The guides were designed to comprehensively capture all predefined qualitative themes. Each IDI was conducted in Vietnamese and lasted approximately 25–30 minutes.” |
| Data analysis: Please elaborate on how you performed the qualitative data analysis. In one place, you had mentioned that the interview guide was developed based on some pre-determined themes. Then, what were they, and how did you analyze the data? | We added three groups of qualitative pre-determined themes in the Qualitative themes section as mentioned above.  We clarified the analysis in more detail as follow:  “All IDIs were audio-recorded, transcribed verbatim in Vietnamese, and checked for accuracy against the original recordings. Data were analyzed using a thematic analysis approach. An initial coding framework was developed based on the predefined themes in the interview guides, and was iteratively refined to incorporate emerging themes from the data.  Two members of the research team independently coded a subset of transcripts to ensure consistency, and discrepancies were discussed and resolved through consensus. The finalized coding framework was then applied to all transcripts. Codes were organized into categories and overarching themes using Mindmap software to facilitate visualization and comparison across participants.  Qualitative findings were used to triangulate, contextualize, and enrich the quantitative results, providing a more comprehensive understanding of factors associated with TB-stigma.” |
| Results: You have a theme mentioned as ‘Attitudes and behaviors of healthcare workers in the Pneumonia Disease Department, Hue Central Hospital’. Do you have any quotes from healthcare workers to support the findings? | We did not have any quote from healthcare workers about this theme since it was discussed with TB patients only. We provided additional information about qualitative themes as mentioned above. |
| **4. Reviewer 3** | |
| ***Introduction Section:*** | |
| What is the author's research question? The research question presented in the paper is not commensurate with the results and discussion provided. | We agree with the reviewer that in our first manuscript, the research question was not presented. We have revised, rewritten and added the research question into the Introduction to better align with our results and discussion.  The research question now explicitly encompasses not only the estimation of stigma prevalence but also the underlying mechanisms such as social prestige, socioeconomic paradoxes, and structural barriers that emerged as central themes in our discussion. This adjustment ensures that the study’s objectives are commensurate with the comprehensive psychosocial and systemic analysis provided in the later sections of the manuscript. |
| It is necessary to define what stigma is and the factors that influence it. In the context of pulmonary TB, does stigma exacerbate the disease, and what are the health issues it causes? In summary, the author needs to supplement the theoretical framework of stigma, including definitions, influencing factors, assessment methods, and its implications for pulmonary TB. | We thank the reviewer for this insightful suggestion. We have significantly strengthened the Introduction by including a definition of stigma and incorporating a dedicated theoretical framework for TB stigma.  Furthermore, we have elaborated on the influencing factors of stigma, distinguishing between individual socioeconomic characteristics and structural factors inherent in tertiary-level specialized care. This addition explains how cultural perceptions of TB as a “disease of poverty” interact with the high visibility of national-tier hospitals to shape patients’ stigmatizing experiences. |
| Lines 90 to 94: The author mentions Hue Central Hospital as an introductory lead for the study site, but there is a complete lack of reference information regarding incidence rates, the number of pulmonary TB patients currently under management, etc. A justification for selecting this study site should be added. | We thank the reviewer for the suggestion to provide more specific data on the study site within the Introduction. We have updated the manuscript accordingly:   - In the Introduction: We have incorporated specific clinical statistics to justify the study setting. In 2023, the Department of Pulmonary Diseases at Hue Central Hospital handled 3,601 TB-related consultations, including 912 inpatients diagnosed with pulmonary TB and 341 referrals to the DOTS program (Reference 14). These figures underscore the high clinical burden and the important role that Hue Central Hospital plays in TB management within the national and regional healthcare networks. - In the Methods (Study site section): We have added a detailed description of the hospital. This provides international readers with the necessary context regarding the hospital's infrastructure and its function as a tertiary referral hub in the Central and Highland of Vietnam.   We hope that these additions clarify the rationale for selecting this site and its representativeness for the study population. |
| ***Materials and Methods Section:*** |  |
| Lines 102, 103: Revise 6/2023 to June 2023, 11/2024 to November 2024; apply similar changes to 25/05/2024 and 15/07/2024. | We thanked the reviewer for this comment and revised accordingly |
| Lines 105 to 108: Is the study population newly diagnosed pulmonary TB patients or those currently undergoing treatment? Were patients with pulmonary TB and concurrent extrapulmonary TB excluded? | Thank you for this important clarification. For the quantitative component, the study population comprised inpatients with pulmonary tuberculosis (pTB) who were admitted to the Department of Pulmonary Diseases at Hue Central Hospital during the study period. These patients were at different stages of treatment, including both newly diagnosed individuals and those currently undergoing treatment; the study did not restrict inclusion to newly diagnosed cases only.  Regarding comorbid forms of tuberculosis, patients were eligible if they had a diagnosis of pulmonary TB. Individuals with concurrent extrapulmonary TB were not explicitly excluded, provided that pulmonary TB was present and they were hospitalized in the Department of Pulmonary Diseases and met all other inclusion criteria. |
| Lines 109 to 113: Why did the study not conduct in-depth interviews with the patients' relatives, as this could also be related to patient stigma alongside doctors and nurses? Did the in-depth interview participants directly treat the 200 patients in the quantitative study? | Thank you for this valuable comment. For the qualitative component, participants were purposively selected from two groups: (1) pTB patients who had participated in the quantitative survey, and (2) healthcare providers (physicians and nurses) who had worked for at least three months in the Department of Pulmonary Diseases at Hue Central Hospital and were directly involved in the clinical care of pTB patients.  We acknowledge that patients’ relatives may also play an important role in shaping stigma. However, the qualitative component was designed to focus on patients’ perceived stigma and their interactions with the healthcare system. Therefore, we prioritized interviewing patients and healthcare providers as the two groups most directly involved in care and stigma-related experiences within the hospital context. The inclusion of relatives was beyond the scope of the current study but is an important consideration for future research.  Regarding the second question, the healthcare providers who participated in the in-depth interviews were working in the same department where the quantitative study was conducted and were involved in the care of pTB patients. However, they were not necessarily directly responsible for treating all 200 patients included in the quantitative sample. They might provide care for some of these 200 pTB patients. |
| Lines 120-121: Information on the number of patients and average treatment duration should be omitted here and moved to the Introduction | Thank you for this helpful suggestion. We agree that detailed contextual information on patient volume and average length of stay may be more appropriate in the Introduction. Accordingly, we have revised the manuscript by moving the information on the average number of hospitalized pTB patients per week and the mean duration of hospitalization to the Introduction section to better contextualize the study setting.  In the Methods section, we have streamlined the description to focus on the sampling strategy and recruitment process, clarifying that all eligible pTB inpatients admitted to the Department of Pulmonary Diseases at Hue Central Hospital during the data collection period (May 25 to July 15, 2024) were approached, resulting in a final sample of 200 participants. |
| Line 123: Correct the error from 25/05/2024 to 15/07/2024 | We revised the date accordingly |
| Lines 130 to 146: What is the VTSS cut-off score for stigma? | Thank you for your question. In this study, the cut-off score for stigma based on the VTSS was determined using the sample mean. The mean VTSS score was 21 (SD = 8), and this value was used as the threshold. Participants with VTSS scores above 21 were classified as experiencing stigma. |
| Line 131: Reference No. 27 is from Brazil, while Reference No. 26 is from Vietnam. Please review the citations. | We thanked reviewer for pointing this mistake, we revised the citations accordingly |
| Line 142: Spelling error. | Thank you for your comment. We have revised and made it corrected, |
| Line 153: 5,000,000 VND? This should be expressed in USD for comparison across studies. Additionally, the total annual income should be used to calculate the cost of treatment. | Thank you for your comment. We have converted the amount of 5,000,000 VND into USD189.6 for comparison across studies. |
| Lines 157 to 162: What is the cut-off score for good versus poor knowledge? In Table 1, the author only mentions the level of knowledge | Thank you for your question. The cut-off point used to categorize knowledge levels was based on the mean knowledge score of the study sample. Specifically, a score of 7.04 was used as the threshold: participants with scores ≥7.04 were classified as having good knowledge, while those with scores <7.04 were classified as having poor knowledge. |
| Lines 172-173: The author states that data were collected by "two staff members from the General Planning Department and two nurses from the Department of Tropical Diseases at Hue Central Hospital." All four individuals are from a different department than the study site (Pulmonary Disease Department). The author needs to clarify: who was in the research team, how were tasks assigned, who conducted the in-depth interviews? Who determined that a patient experienced stigma? Are all four of these individuals included in the author list? If not, please clarify potential conflicts of interest, if any. | Thank you for this important request for clarification.  The data collection process was led by the principal investigator, who was the first author of this paper, LTVT, one staff member from the General Planning Department and two nurses from the Department of Tropical Diseases at Hue Central Hospital. These individuals were intentionally selected from departments other than the Department of Pulmonary Diseases to minimize potential bias and reduce perceived pressure on patients.  All four data collectors were responsible for participant recruitment and administration of the quantitative questionnaires. For the qualitative component, in-depth interviews (IDIs) were conducted by LTVT only (the principal investigator), who had prior training and experience in qualitative research methods. The remaining team members supported logistics and coordination but did not conduct the IDIs.  Patients experiencing stigma were identified based on their responses to the structured VTSS, using predefined criteria and scoring procedures established prior to data collection. These results were then used to purposely select participants for the qualitative interviews.  Except for LTVT, the other three data collectors are not included in the author list. Authorship was determined in accordance with standard authorship criteria, based on substantial contributions to study conception and design, data analysis and interpretation, and manuscript development. The data collectors who are not listed as authors were involved solely in data collection activities and did not have a role in data analysis or interpretation. There are no conflicts of interest to declare. |
| Lines 183-184: In lines 125-126, the author mentions an in-depth interview with a Hospital Management Board member, whereas in this section, the author mentions interviewing a Department Head. This shows a lack of consistency and requires clarification. Furthermore, please be consistent in using "physician" or "doctor," and whether the research unit is the "Pulmonary Disease Department" or "Department of Pulmonary Disease." | Thank you for highlighting these inconsistencies. We apologize for the lack of clarity in terminology.  We have carefully reviewed and revised the manuscript to ensure consistency throughout. Specifically, the interview participant is now consistently referred to as the Head of the Department, the term “doctor” is used uniformly instead of “physician,” and the study setting is consistently described as the Department of Pulmonary Disease.  All relevant sections of the manuscript have been updated accordingly. |
| Line 190: Which version of EpiData was used? | Thank you for your question. Data entry was performed using EpiData version 3.0, and this information has now been specified in the Methods section of the revised manuscript. |
| Lines 198 to 208: Were patients identified as having stigma referred to a psychiatrist for counseling? | Thank you for this important question. In this study, stigma was assessed for research purposes using a structured questionnaire, and participants were not clinically diagnosed with a mental health disorder. Therefore, routine referral to a psychiatrist was not part of the study protocol.  However, ethical considerations were carefully addressed. Participants who expressed distress or a need for psychological support during the interviews were advised to seek counseling services available at the hospital and were referred to appropriate healthcare providers upon request. |
| ***Results Section*** |  |
| Line 234: Fig. 1: Re-evaluate the chart presentation; percentage values overlap, making it difficult to follow. | Thank you for this helpful suggestion. We agree that the original figure was difficult to interpret due to overlapping percentage values. Accordingly, we have replaced Fig. 1 with a table format to improve clarity and readability. |
| Could the author specify which in-depth interview participant (coded starting with PVS) served as the Hospital Management Board member? | Thank you for this helpful suggestion. “PVS” is the Vietnamese abbreviation for in-depth interviews, equivalent to “IDI” (in-depth interview) in English. We have revised the manuscript accordingly and replaced all instances of “PVS” with “IDI” to ensure consistency. |
| ***Discussion Section*** |  |
| Line 356: Is the "Department of Lung Disease" the unit where the study was conducted, or is it another unit? The author needs to standardize terminology. | We sincerely apologize for the inconsistency in terminology. We can confirm that the study was conducted in a single unit. To ensure clarity and standardization throughout the manuscript, we have revised all instances to "Department of Pulmonary Diseases". We have cross-checked the Abstract, Methodology, and Discussion sections to ensure that this terminology is used consistently. |
| Lines 356 to 377: The author needs to rewrite this section; it contains the most critical information of the article, yet it is poorly handled. What is the significance of the 43.5% stigma prevalence? As the author presented, this is lower than in studies at other locations - what explains this difference? | Thank you for your comment. We totally agree with this and have rewritten our discussion for this results, specifically as follow:   - The “institutional buffer" hypothesis: We explain that the lower prevalence compared to community-based studies in Cambodia (Teo et al., 2020) and Indonesia (Megawati et al., 2024) is likely due to the "institutional buffer" provided by specialized tertiary care at a national-level hospital, which mitigates overt acts of discrimination. - Internalization vs. Enactment: We clarified that while external discrimination (enacted stigma) is lower, self-stigma (27.5% guilt over family burden) remains profoundly high, suggesting that clinical cure does not equate to social reintegration. - We integrated qualitative insights to show that at this tertiary level, positive patient-provider interactions act as a countervailing force against institutional stigma, distinguishing our findings from community-level reports. |
| Line 431: "department of lung disease"? Have the study's limitations been adequately acknowledged?  Numerous issues ranging from the study population and sampling methods to data collection procedures influence the results of this study. | We thank the reviewer for indicating the grammar typing and has replaced “department of lung disease” into “Department of Pulmonary Diseases” for consistency throughout the manuscript.  Furthermore, we have significantly expanded the Limitations section to acknowledge potential biases related to the study population, the convenience sampling method at a single tertiary center, and the possibility of social desirability bias during data collection. We have also discussed how these factors might influence the generalizability of our findings." |
| **5. Reviewer 4** | |
| ***Language and Writing Quality*** | |
| The manuscript contains several grammatical errors. Examples of specific errors  - Line 40: “pulmorary tubeculosis” should read pulmonary tuberculosis  - Line 151: “Christinality” should be Christianity | We sincerely apologize for the grammatical errors. We have made them corrected and have checked throughout the documents to correct all the grammatical errors. |
| ***Structural Completeness of Sections*** | |
| Abstract: Key statistical measures such as odds ratios and confidence intervals are not reported. | We thank the reviewer for this comment and we have added key statistical measures (OR, 95%CI) into the abstract. |
| Introduction: The research gap is weakly articulated. | Thank you for this important comment. We have revised the introduction and added the research gaps into the introduction. You may find our responses to other reviewers above for our revision of the Introduction. |
| Methods - The sampling strategy is insufficiently described. - It is unclear whether sampling was consecutive, random, or convenience-based. | Thank you for this important comment. We have revised the Methods section to clarify the sampling strategy.  “For the quantitative component, we employed a consecutive sampling approach. Specifically, all eligible pTB inpatients admitted to the Department of Pulmonary Disease at Hue Central Hospital during the data collection period were approached and invited to participate until the target sample size was reached.  For the qualitative component, we used purposive sampling to select participants from two groups: (1) pTB patients who had participated in the quantitative survey (with variation in stigma experiences), and (2) healthcare providers (doctors and nurses) working in the same department.”  These details have now been explicitly stated in the revised manuscript. |
| Discussion - Interpretation remains largely descriptive. The manuscript does not describe how qualitative themes were integrated with quantitative results.  - The manuscript does not sufficiently explore mechanisms underlying stigma, such as cultural beliefs, or institutional factors. | We thank the reviewer for these valuable comments.  We have fundamentally restructured the Discussion to move beyond descriptive reporting and provide a rigorous, integrated interpretation of our mixed-methods data:   - Integration of themes: We have explicitly linked quantitative findings (e.g., higher income and stigma) with qualitative themes (e.g., status loss) to explain the underlying mechanisms of stigma. - Mechanistic exploration: We now explore how institutional fragmentation and healthcare tiering influence stigma prevalence, contrasting our results with community-based studies in other contexts. - Institutional dimension: We have engaged with the literature on healthcare professional stigma to discuss the multi-dimensional role of hospital staff as both potential sources of stigma and vital psychosocial buffers. |
| Limitations: The limitations section is incomplete and fails to acknowledge several important epidemiological threats to validity. | We are thankful for the reviewer’s comment. We have expanded the Limitations section to acknowledge key threats to validity, including selection bias, social desirability bias, and the constraints of a hospital-based cross-sectional design. We also include another limitation “no in-depth interview was conducted with patients’ family members/relatives” |
| Recommendation: Authors should complete the missing pieces of information. | Thank you for your suggestion. We have re-written the recommendation to complete the missing pieces of information. |
| ***Validity of the Measurement Instrument*** | |
| Authors should report Cronbach’s alpha values for the stigma scale in the current sample. | Thank you for this important suggestion. We have now calculated and reported the internal consistency of the stigma scale in the study sample. The Cronbach’s alpha coefficient was 0.82, indicating good reliability. This information has been added to the Methods section of the revised manuscript. |
| ***Evaluation of Tables and Figures*** | |
| Table 1“Marriage” → should be Marital status | Thank you for pointing this out. We have revised Table 2 by replacing “Marriage” with “Marital status” to ensure accurate and appropriate terminology. |
| ***Epidemiological Bias Assessment*** |  |
| A rigorous epidemiological evaluation requires consideration of four key threats to validity.  *Selection Bias:* The study recruited participants only from hospitalized pulmonary tuberculosis patients in one hospital, which may introduce facility-based selection bias (Berkson’s bias). This occurs because the sample includes only patients who are admitted and already engaged with hospital care, while TB patients treated elsewhere or those avoiding care are excluded. These groups may experience different forms or levels of stigma. However, the study title clearly specifies that the focus is on hospitalized TB patients. Therefore, the findings are appropriate for this specific population but may not be generalizable to all TB patients in the broader community.  *Information Bias:* Although the researchers acknowledge social desirability bias, there might also be interviewer bias. This occurs when participants modify their responses due to the presence or perceived expectations of the interviewer. In this study, data were collected by nurses from the same hospital, which could have influenced patients to provide answers they thought were acceptable or favorable. Such a setting increases the likelihood that responses were shaped by the interviewer’s role or authority. Therefore, interviewer bias is a plausible limitation that should be considered alongside social desirability bias.  *Confounding:* The regression model includes several demographic variables, but the process for confounder selection is unclear. Potential confounders not included include: • duration of illness  • treatment phase  • family history of TB  • psychological distress  • prior stigma exposure  Without adjustment for these variables, observed associations may be biased.  *Reverse Causality:* Because the study uses a cross-sectional design, temporal direction cannot be established.  Example: • Higher stigma among unmarried participants may not reflect causation. Stigma itself may influence relationship stability or marital prospects. This possibility is not discussed in the manuscript. | We are deeply grateful for the reviewer’s rigorous epidemiological assessment of our study. We have revised the Limitations section to explicitly address these four critical threats to validity:   - Selection bias (Berkson’s Bias): We now acknowledge that recruiting only hospitalized patients at a tertiary hospital may exclude those with different stigma experiences in the community, potentially biasing our prevalence estimates. - Information bias (Interviewer & Social Desirability): We have clarified that the involvement of hospital nurses in data collection, combined with the cultural emphasis on family reputation/status loss, may have influenced patients to provide more socially acceptable answers. - Confounding: We have admitted the omission of clinical variables (e.g., treatment phase, illness duration) and psychological distress as a limitation that may lead to residual confounding in our regression analysis. - Qualitative gaps: We have included the absence of interviews with patients' relatives as a limitation, noting that this restricts our holistic understanding of familial stigma. - Reverse causality: We have addressed the temporal ambiguity of our cross-sectional design, specifically discussing how stigma can be both a cause and a consequence of social factors like marital instability |
| Mixed-Methods Integration: The qualitative component is valuable but insufficiently integrated with quantitative findings. Current structure appears sequential 1. quantitative results  2. qualitative quotations  However, the analysis does not demonstrate: • how qualitative themes explain statistical associations • or whether qualitative findings were used to generate hypotheses Recommendation A stronger mixed-methods design would explicitly connect themes with quantitative results. | We thank the reviewer for this important comment. We already state the limitation in response to this comment.   - Methodological transparency: In the Limitations section, we have clarified our mixed-methods approach. While we utilized qualitative data to explain the "how" and "why" behind the quantitative trends, we acknowledge that further integration (such as using qualitative data for hypothesis generation) could be an avenue for future research. |
| The study addresses a relevant issue in tuberculosis care, particularly in contexts where stigma continues to undermine treatment engagement. However, major revision required before reconsideration for publication. | Thank you for your valuable comments. We have addressed your comment and all other comments from the other reviewers to make major revisions for the manuscript. |
| Others | Based on reviewers’ suggestion, we also undertook some other revisions as follows:   - Title revision: We have updated the title to “Perceived stigma among hospitalized pulmonary tuberculosis patients in Hue Central Hospital: a 2024 mixed-method study in Vietnam” to reflect the study’s focus more accurately. - Terminology consistency: We have systematically updated all terminologies throughout the manuscript, transitioning from the general term ‘stigma’ to the more specific and theoretically grounded term ‘perceived stigma’. - Conceptual clarification: We have incorporated a definition of perceived stigma in the Introduction, distinguishing between its anticipated and internalized dimensions. - Methodological Refinement: In the Methods section, we have explicitly operationalized the concept of perceived stigma. We clarified how this multidimensional construct was measured using the Van Rie TB Stigma Scale (VTSS) and how our qualitative interview guide was designed to further explore the nuances of patients’ internal perceptions and fears. |
