# Supplementary material for: Perceived stigma among hospitalized pulmonary tuberculosis patients in Hue Central Hospital: A 2024 mixed method study in Vietnam
Source: PLOS Glob Public Health. 2026 Jul 13;6(7):e0006752. doi: 10.1371/journal.pgph.0006752 (PMC13362144; doi:10.1371/journal.pgph.0006752)
Supplement: Peer review history

# Peer review: Decision Letter 0

**DOI:** 10.1371/journal.pgph.0006752.r001

**Authors:** Graeme Hoddinott

31 Mar 2026

PGPH-D-26-00084

Stigma of hospitalized pulmonary tuberculosis patients in Hue Central Hospital: a 2024 mixed method study in Vietnam

PLOS Global Public Health

Dear Dr. Nguyen,

Thank you for submitting your manuscript to PLOS Global Public Health. After careful consideration, we feel that it has merit but does not fully meet PLOS Global Public Health’s publication criteria as it currently stands. Therefore, we invite you to submit a revised version of the manuscript that addresses the points raised during the review process.

==============================

EDITOR:

In my view, the reviewers have suggested a number of revisions that will strengthen the manuscript. I trust that you receive these in the spirit of encouragement.

==============================

Please submit your revised manuscript by May 15 2026 11:59PM. If you will need more time than this to complete your revisions, please reply to this message or contact the journal office at globalpubhealth@plos.org. When you're ready to submit your revision, log on to https://www.editorialmanager.com/pgph/ and select the 'Submissions Needing Revision' folder to locate your manuscript file.

Please include the following items when submitting your revised manuscript:

- A letter that responds to each point raised by the editor and reviewer(s). You should upload this letter as a separate file labeled 'Response to Reviewers'.
- A marked-up copy of your manuscript that highlights changes made to the original version. You should upload this as a separate file labeled 'Revised Manuscript with Track Changes'.
- An unmarked version of your revised paper without tracked changes. You should upload this as a separate file labeled 'Manuscript'.

Guidelines for resubmitting your figure files are available below the reviewer comments at the end of this letter.

As the corresponding author, your ORCID iD is verified in the submission system and will appear in the published article. PLOS supports the use of ORCID, and we encourage all coauthors to register for an ORCID iD and use it as well. Please encourage your coauthors to verify their ORCID iD within the submission system before final acceptance, as unverified ORCID iDs will not appear in the published article. *Only*  the individual author can complete the verification step; PLOS staff *cannot*  verify ORCID iDs on behalf of authors.

We look forward to receiving your revised manuscript.

Kind regards,

Graeme Hoddinott, Ph.D

Academic Editor

PLOS Global Public Health

Journal Requirements:

1. We note that you have indicated that there are restrictions to data sharing for this study. For studies involving human research participant data or other sensitive data, we encourage authors to share de-identified or anonymized data. However, when data cannot be publicly shared for ethical reasons, we allow authors to make their data sets available upon request. For information on unacceptable data access restrictions, please see http://journals.plos.org/globalpublichealth/s/data-availability#loc-unacceptable-data-access-restrictions.

Before we proceed with your manuscript, please address the following prompts:

a) If there are ethical or legal restrictions on sharing a de-identified data set, please explain them in detail (e.g., data contain potentially identifying or sensitive patient information, data are owned by a third-party organization, etc.) and who has imposed them (e.g., a Research Ethics Committee or Institutional Review Board, etc.). Please also provide contact information for a data access committee, ethics committee, or other institutional body to which data requests may be sent.

b) If there are no restrictions, please upload the minimal anonymized data set necessary to replicate your study findings to a stable, public repository and provide us with the relevant URLs, DOIs, or accession numbers. Please see http://www.bmj.com/content/340/bmj.c181.long for guidelines on how to de-identify and prepare clinical data for publication. For a list of recommended repositories, please see https://journals.plos.org/globalpublichealth/s/recommended-repositories. You also have the option of uploading the data as Supporting Information files, but we would recommend depositing data directly to a data repository if possible.

Please update your Data Availability statement in the submission form accordingly.

2. Please provide separate main figure files in .tif or .eps format only and ensure that all files are under our size limit of 10MB.

For more information about how to convert your figure files please see our guidelines: https://journals.plos.org/globalpublichealth/s/figures

3. We note that the ethics approval documents should not be published with the paper and are for internal information only. Please change the file type to 'Other' to ensure that they are not published.

4. If the reviewer comments include a recommendation to cite specific previously published works, please review and evaluate these publications to determine whether they are relevant and should be cited. There is no requirement to cite these works unless the editor has indicated otherwise.

Additional Editor Comments (if provided):

[Note: HTML markup is below. Please do not edit.]

Reviewers' comments:

Reviewer's Responses to Questions

**Comments to the Author**

1. Does this manuscript meet PLOS Global Public Health’s publication criteria? Is the manuscript technically sound, and do the data support the conclusions? The manuscript must describe methodologically and ethically rigorous research with conclusions that are appropriately drawn based on the data presented.

Reviewer #1: Yes

Reviewer #2: Partly

Reviewer #3: Yes

Reviewer #4: Partly

**********

2. Has the statistical analysis been performed appropriately and rigorously?

Reviewer #1: Yes

Reviewer #2: Yes

Reviewer #3: Yes

Reviewer #4: Yes

**********

3. Have the authors made all data underlying the findings in their manuscript fully available (please refer to the Data Availability Statement at the start of the manuscript PDF file)?

The PLOS Data policy requires authors to make all data underlying the findings described in their manuscript fully available without restriction, with rare exception. The data should be provided as part of the manuscript or its supporting information, or deposited to a public repository. For example, in addition to summary statistics, the data points behind means, medians and variance measures should be available. If there are restrictions on publicly sharing data—e.g. participant privacy or use of data from a third party—those must be specified.

Reviewer #1: Yes

Reviewer #2: Yes

Reviewer #3: Yes

Reviewer #4: Yes

**********

4. Is the manuscript presented in an intelligible fashion and written in standard English?

PLOS Global Public Health does not copyedit accepted manuscripts, so the language in submitted articles must be clear, correct, and unambiguous. Any typographical or grammatical errors should be corrected at revision, so please note any specific errors here.

Reviewer #1: No

Reviewer #2: Yes

Reviewer #3: Yes

Reviewer #4: Yes

**********

5. Review Comments to the Author

Please use the space provided to explain your answers to the questions above. You may also include additional comments for the author, including concerns about dual publication, research ethics, or publication ethics. (Please upload your review as an attachment if it exceeds 20,000 characters)

Reviewer #1: Reviewer Comments

This manuscript investigates tuberculosis-related stigma among hospitalized pulmonary tuberculosis patients in Hue Central Hospital in Vietnam using a mixed-methods design that combines quantitative assessment with qualitative interviews. The topic addressed by the authors is highly relevant for global tuberculosis control, as stigma continues to represent a major barrier to early diagnosis, treatment adherence, and social reintegration of patients. The integration of the Van Rie Tuberculosis Stigma Scale with qualitative narratives offers an important opportunity to capture both measurable and experiential dimensions of stigma among pulmonary tuberculosis patients. The study reports that 43.5% of hospitalized pulmonary tuberculosis patients experienced stigma, with high levels of selective disclosure, avoidance behaviors, and perceived social distancing. These findings contribute to the growing literature demonstrating that tuberculosis-related stigma remains a persistent psychosocial burden even within clinical settings where patients are receiving treatment.

However, several conceptual and methodological issues should be addressed in order to strengthen the manuscript. First, the introduction would benefit from a stronger integration with recent epidemiological literature on tuberculosis vulnerability and healthcare access. Tuberculosis-related stigma does not occur in isolation but is closely linked to structural determinants such as diagnostic practices, health system capacity, and the social context of vulnerable populations. Recent studies have highlighted how diagnostic inequalities and structural barriers may influence both the clinical course of tuberculosis and the psychosocial experiences of affected patients. In this regard, the authors may consider integrating recent studies examining tuberculosis epidemiology, diagnostic access, and healthcare utilization, including work demonstrating diagnostic shifts and testing gaps in tuberculosis care. For example, the study by Çil et al. examining diagnostic shifts in extrapulmonary tuberculosis during the COVID-19 period highlights how health system disruptions and population vulnerabilities can influence tuberculosis diagnosis and care pathways (Çil B, Bodur MS, Kabak M. Diagnostic shifts in extrapulmonary tuberculosis during COVID-19: evidence of vulnerability among migrants in a border province. BMC Infectious Diseases. 2026). Similarly, evidence demonstrating underutilization of bacteriological testing in extrapulmonary tuberculosis suggests that diagnostic practices remain inconsistent across healthcare settings and may contribute to delayed diagnosis and social vulnerability among tuberculosis patients (Çil B, Bodur MS, Kabak M. Underutilization of Bacteriological Testing in Extrapulmonary Tuberculosis: A Retrospective Single-center Study from Mardin, Türkiye. Thoracic Research and Practice. 2026). Incorporating such literature would strengthen the conceptual background and provide a broader perspective on structural determinants influencing tuberculosis care.

Second, the manuscript would benefit from clearer conceptual differentiation between different forms of stigma. The study frequently refers to “stigma” as a single construct; however, tuberculosis-related stigma can manifest in multiple forms including perceived stigma, internalized stigma, enacted stigma, and institutional stigma. Several of the behaviors reported in the study appear to reflect internalized or anticipated stigma rather than direct discriminatory experiences. For example, the finding that more than 90% of participants reported intentionally keeping distance from others may partly reflect infection-control awareness rather than stigma alone. Distinguishing these dimensions would allow a more nuanced interpretation of the findings and would align the study with current conceptual frameworks used in tuberculosis stigma research.

Third, the classification approach used to define stigma prevalence requires clarification. The authors define stigma as present when the raw score exceeds the standardized summary score (SSraw > SS50). This method differs from commonly used scoring strategies in studies utilizing the Van Rie Tuberculosis Stigma Scale. Additional justification for this threshold should therefore be provided, including references supporting this approach and a discussion of how this classification may influence the reported prevalence of stigma in the study population. Without clarification, the estimate that 43.5% of hospitalized pulmonary tuberculosis patients experience stigma may be difficult to interpret in comparison with other studies.

Another issue concerns the interpretation of socioeconomic findings. The study reports that higher-income patients experienced higher levels of stigma compared with lower-income patients (adjusted OR = 2.01). This finding contradicts much of the existing literature, where stigma is more commonly associated with poverty, limited access to healthcare, and lower socioeconomic status. The discussion should therefore provide a deeper interpretation of this result and consider potential explanations such as sampling bias due to the hospital-based study design, contextual social dynamics, or measurement limitations within the stigma scale.

The qualitative findings provide valuable insights into structural barriers affecting tuberculosis patients, including fragmented referral pathways, difficulties navigating decentralized treatment systems, and financial challenges related to treatment of comorbidities. These structural barriers appear to play a significant role in shaping patients’ psychosocial experiences and may contribute to both treatment delays and increased stigma. Expanding the discussion of health system factors would strengthen the manuscript and align the findings with broader research examining healthcare access and tuberculosis vulnerability.

Finally, the manuscript would benefit from engagement with literature examining stigma among healthcare professionals themselves. Evidence suggests that healthcare workers’ attitudes and perceptions toward tuberculosis patients may influence patient experiences and treatment engagement. For instance, research exploring stigma among healthcare professionals has demonstrated that institutional attitudes can play a critical role in shaping patient experiences and healthcare-seeking behavior (Bodur MS, Çil B. A Research on Healthcare Professionals’ Stigma Towards Tuberculosis Patients. Thoracic Research and Practice. 2025). Integrating such literature would enrich the discussion and highlight the multidimensional nature of tuberculosis-related stigma within healthcare systems.

In summary, the manuscript addresses an important public health issue and provides valuable insights into the psychosocial burden experienced by hospitalized pulmonary tuberculosis patients. The mixed-methods approach and use of a validated stigma scale represent strengths of the study. However, the manuscript would benefit from stronger conceptual framing, clearer methodological justification, deeper interpretation of socioeconomic findings, and broader integration with recent tuberculosis literature addressing diagnostic practices, healthcare access, and stigma dynamics. Addressing these points would significantly strengthen the scientific contribution of the manuscript and enhance its relevance for global tuberculosis control research.

Reviewer #2: Thank you for the opportunity to review the paper titled ‘Stigma of hospitalized pulmonary tuberculosis patients in Hue Central Hospital: a 2024 mixed method study in Vietnam’. Some of my suggestions to improve its rigor are provided below.

Aim of the study: The aim has been mentioned as to estimate the prevalence of stigma and identify factors in the Abstract, while the aim mentioned in the Introduction is to describe the status of stigma and associated factors. Given the mixed methods design, this is acceptable, but make it consistent in both the abstract and the introduction.

Study design, time, and location: Why Hue Central Hospital? Justify the selection of this study setting and briefly describe its location and characteristics, such as facilities and treatments available for TB patients, so that readers understand the context.

Study participants: Who made the participant recruitment, and what was the procedure? Was there any involvement by the hospital staff in recruiting patients for the study, if so, how did you avoid any potential bias?

Sample size and selection of participants: You have conducted 13 in-depth interviews. Did you conduct interviews until data saturation was reached? If so, how did you decide the point of saturation?

Qualitative study themes- You have a sub-heading: ‘qualitative study themes’ which is not clear. No themes are mentioned. You can remove the term ‘themes’ from here.

Data collection tool, technique, and procedure: It seems your questionnaire is interviewer-administered; please clarify.

Qualitative study: You have mentioned that ‘a set of qualitative in-depth interviews (IDI) guides’ were used, which is not clear. How many interview guides were developed and for whom? How long was an interview? What was the language in which you conducted the interviews?

Data analysis: Please elaborate on how you performed the qualitative data analysis. In one place, you had mentioned that the interview guide was developed based on some pre-determined themes. Then, what were they, and how did you analyze the data?

Results: You have a theme mentioned as ‘Attitudes and behaviors of healthcare workers in the Pneumonia Disease Department, Hue Central Hospital’. Do you have any quotes from healthcare workers to support the findings?

Reviewer #3: Comments to the Author

Tuberculosis (TB)-related stigma remains an alarming social issue in low- and middle-income countries (LMICs), including Vietnam. Stigma tends to increase when patients are hospitalized for inpatient treatment at healthcare facilities; therefore, it is essential to obtain perspectives from both patients and healthcare workers on this matter. Research on this topic is highly necessary and holds both scientific and practical significance, as determining the prevalence and identifying associated factors help find solutions suitable for the practical context, with the ultimate goal of enhancing management effectiveness and patients' quality of life.

Overall, the manuscript is relatively well-prepared; however, several points require clarification and revision, as follows:

Introduction Section:

• What is the author's research question? The research question presented in the paper is not commensurate with the results and discussion provided.

• It is necessary to define what stigma is and the factors that influence it. In the context of pulmonary TB, does stigma exacerbate the disease, and what are the health issues it causes? In summary, the author needs to supplement the theoretical framework of stigma, including definitions, influencing factors, assessment methods, and its implications for pulmonary TB.

• Lines 90 to 94: The author mentions Hue Central Hospital as an introductory lead for the study site, but there is a complete lack of reference information regarding incidence rates, the number of pulmonary TB patients currently under management, etc. A justification for selecting this study site should be added.

Materials and Methods Section:

• Lines 102, 103: Revise 6/2023 to June 2023, 11/2024 to November 2024; apply similar changes to 25/05/2024 and 15/07/2024.

• Lines 105 to 108: Is the study population newly diagnosed pulmonary TB patients or those currently undergoing treatment? Were patients with pulmonary TB and concurrent extrapulmonary TB excluded?

• Lines 109 to 113: Why did the study not conduct in-depth interviews with the patients' relatives, as this could also be related to patient stigma alongside doctors and nurses? Did the in-depth interview participants directly treat the 200 patients in the quantitative study?

• Lines 120-121: Information on the number of patients and average treatment duration should be omitted here and moved to the Introduction.

• Line 123: Correct the error from 25/05/2024 to 15/07/2024.

• Lines 130 to 146: What is the VTSS cut-off score for stigma?

• Line 131: Reference No. 27 is from Brazil, while Reference No. 26 is from Vietnam. Please review the citations.

• Line 142: Spelling error.

• Line 153: 5,000,000 VND? This should be expressed in USD for comparison across studies. Additionally, the total annual income should be used to calculate the cost of treatment.

• Lines 157 to 162: What is the cut-off score for good versus poor knowledge? In Table 1, the author only mentions the level of knowledge.

• Lines 172-173: The author states that data were collected by "two staff members from the General Planning Department and two nurses from the Department of Tropical Diseases at Hue Central Hospital." All four individuals are from a different department than the study site (Pulmonary Disease Department). The author needs to clarify: who was in the research team, how were tasks assigned, who conducted the in-depth interviews? Who determined that a patient experienced stigma? Are all four of these individuals included in the author list? If not, please clarify potential conflicts of interest, if any.

• Lines 183-184: In lines 125-126, the author mentions an in-depth interview with a Hospital Management Board member, whereas in this section, the author mentions interviewing a Department Head. This shows a lack of consistency and requires clarification. Furthermore, please be consistent in using "physician" or "doctor," and whether the research unit is the "Pulmonary Disease Department" or "Department of Pulmonary Disease."

• Line 190: Which version of EpiData was used?

• Lines 198 to 208: Were patients identified as having stigma referred to a psychiatrist for counseling?

Results Section:

• Line 234: Fig. 1: Re-evaluate the chart presentation; percentage values overlap, making it difficult to follow.

• Could the author specify which in-depth interview participant (coded starting with PVS) served as the Hospital Management Board member?

Discussion Section:

• Line 356: Is the "Department of Lung Disease" the unit where the study was conducted, or is it another unit? The author needs to standardize terminology.

• Lines 356 to 377: The author needs to rewrite this section; it contains the most critical information of the article, yet it is poorly handled. What is the significance of the 43.5% stigma prevalence? As the author presented, this is lower than in studies at other locations—what explains this difference?

• Line 431: "department of lung disease"? Have the study's limitations been adequately acknowledged? Numerous issues ranging from the study population and sampling methods to data collection procedures influence the results of this study.

Reviewer #4: Peer Review Report

Manuscript title: Stigma of Hospitalized Pulmonary Tuberculosis Patients in Hue Central Hospital: A 2024 Mixed-Methods Study in Vietnam

Overall Evaluation

This manuscript examines tuberculosis-related stigma among hospitalized pulmonary tuberculosis patients using a mixed-methods design combining quantitative measurement and qualitative interviews. The topic is highly relevant for tuberculosis control, particularly in low- and middle-income countries where stigma can delay care-seeking, reduce treatment adherence, and worsen health outcomes. The attempt to combine quantitative analysis with qualitative narratives is commendable and potentially strengthens the explanatory value of the findings.

However, the manuscript currently requires substantial revision before it can meet publication standards. Major concerns relate to clarity of language, measurement validity, statistical reporting, and incomplete acknowledgment of epidemiological biases. In addition, several typographical errors reduce the scientific rigor of the manuscript.

The comments below provide a detailed assessment.

1. Language and Writing Quality

The manuscript contains several grammatical errors.

Examples of specific errors

• Line 40: “pulmorary tubeculosis” should read pulmonary tuberculosis

• Line 151: “Christinality” should be Christianity

Recommendation

The manuscript requires professional language editing to improve clarity, grammar, and consistency across sections.

2. Structural Completeness of Sections

Abstract

Strengths

• Clearly states the study design.

• Identifies the primary research topic and study population.

Weaknesses

• Key statistical measures such as odds ratios and confidence intervals are not reported.

Introduction

Strengths

• Provides general global context on tuberculosis stigma.

Weaknesses

• The research gap is weakly articulated.

Methods

Strengths

• Mixed-methods design is mentioned.

• Ethical approval is reported.

Weaknesses

• The sampling strategy is insufficiently described.

• It is unclear whether sampling was consecutive, random, or convenience-based.

Results

Strengths

• Descriptive and regression results are presented.

Discussion

Strengths

• The study compares results with findings from other studies.

Weaknesses

• Interpretation remains largely descriptive. The manuscript does not describe how qualitative themes were integrated with quantitative results.

• The manuscript does not sufficiently explore mechanisms underlying stigma, such as cultural beliefs, or institutional factors.

Limitations

The limitations section is incomplete and fails to acknowledge several important epidemiological threats to validity.

Recommendation

Authors should complete the missing pieces of information.

3. Validity of the Measurement Instrument

The study uses the Van Rie Tuberculosis Stigma Scale, which is widely used in tuberculosis stigma research.

Strengths

• The scale has been applied and validated in multiple settings.

• The manuscript cites a Vietnamese validation study.

Missing information

The manuscript does not report key psychometric properties for the current sample such as internal consistency (Cronbach’s alpha). These are standard reporting elements when applying a validated instrument in a new sample.

Recommendation

Authors should report Cronbach’s alpha values for the stigma scale in the current sample.

4. Evaluation of Tables and Figures

Table 1

• “Marriage” → should be Marital status

5. Epidemiological Bias Assessment

A rigorous epidemiological evaluation requires consideration of four key threats to validity.

5.1 Selection Bias

The study recruited participants only from hospitalized pulmonary tuberculosis patients in one hospital, which may introduce facility-based selection bias (Berkson’s bias). This occurs because the sample includes only patients who are admitted and already engaged with hospital care, while TB patients treated elsewhere or those avoiding care are excluded. These groups may experience different forms or levels of stigma. However, the study title clearly specifies that the focus is on hospitalized TB patients. Therefore, the findings are appropriate for this specific population but may not be generalizable to all TB patients in the broader community.

5.2 Information Bias

Although the researchers acknowledge social desirability bias, there might also be interviewer bias. This occurs when participants modify their responses due to the presence or perceived expectations of the interviewer. In this study, data were collected by nurses from the same hospital, which could have influenced patients to provide answers they thought were acceptable or favorable. Such a setting increases the likelihood that responses were shaped by the interviewer’s role or authority. Therefore, interviewer bias is a plausible limitation that should be considered alongside social desirability bias.

5.3 Confounding

The regression model includes several demographic variables, but the process for confounder selection is unclear.

Potential confounders not included include:

• duration of illness

• treatment phase

• family history of TB

• psychological distress

• prior stigma exposure

Without adjustment for these variables, observed associations may be biased.

5.4 Reverse Causality

Because the study uses a cross-sectional design, temporal direction cannot be established.

Example:

• Higher stigma among unmarried participants may not reflect causation.

Stigma itself may influence relationship stability or marital prospects.

This possibility is not discussed in the manuscript.

Recommendation

All these limitations should be addressed or at least be mentioned in the limitations of the study.

6. Mixed-Methods Integration

The qualitative component is valuable but insufficiently integrated with quantitative findings.

Current structure appears sequential

1. quantitative results

2. qualitative quotations

However, the analysis does not demonstrate:

• how qualitative themes explain statistical associations

• or whether qualitative findings were used to generate hypotheses

Recommendation

A stronger mixed-methods design would explicitly connect themes with quantitative results.

Final Recommendation

The study addresses a relevant issue in tuberculosis care, particularly in contexts where stigma continues to undermine treatment engagement. However, major revision required before reconsideration for publication.

**********

6. PLOS authors have the option to publish the peer review history of their article (what does this mean?). If published, this will include your full peer review and any attached files.

**Do you want your identity to be public for this peer review?**  If you choose “no”, your identity will remain anonymous but your review may still be made public.

For information about this choice, including consent withdrawal, please see our Privacy Policy.

Reviewer #1: No

Reviewer #2: **Yes:**  Nadeeka Rathnayake

Reviewer #3: No

Reviewer #4: No

**********

[NOTE: If reviewer comments were submitted as an attachment file, they will be attached to this email and accessible via the submission site. Please log into your account, locate the manuscript record, and check for the action link "View Attachments". If this link does not appear, there are no attachment files.]

-->Figure Resubmissions:

While revising your submission, we strongly recommend that you use PLOS’s NAAS tool (https://ngplosjournals.pagemajik.ai/artanalysis) to test your figure files. NAAS can convert your figure files to the TIFF file type and meet basic requirements (such as print size, resolution), or provide you with a report on issues that do not meet our requirements and that NAAS cannot fix.

After uploading your figures to PLOS’s NAAS tool - https://ngplosjournals.pagemajik.ai/artanalysis, NAAS will process the files provided and display the results in the "Uploaded Files" section of the page as the processing is complete. If the uploaded figures meet our requirements (or NAAS is able to fix the files to meet our requirements), the figure will be marked as "fixed" above. If NAAS is unable to fix the files, a red "failed" label will appear above. When NAAS has confirmed that the figure files meet our requirements, please download the file via the download option, and include these NAAS processed figure files when submitting your revised manuscript.

Attachment Submitted filename: Comments- Plos Global Public Health.docx

# Author comment: Author response to Decision Letter 1

**DOI:** 10.1371/journal.pgph.0006752.r002

30 May 2026

Attachment Submitted filename: 4. Response_to_reviewers.docx

# Peer review: Decision Letter 1

**DOI:** 10.1371/journal.pgph.0006752.r003

**Authors:** Graeme Hoddinott, Graeme Hoddinott

10 Jun 2026

Perceived stigma among hospitalized pulmonary tuberculosis patients in Hue Central Hospital: a 2024 mixed method study in Vietnam

PGPH-D-26-00084R1

Dear MS Nguyen,

We are pleased to inform you that your manuscript 'Perceived stigma among hospitalized pulmonary tuberculosis patients in Hue Central Hospital: a 2024 mixed method study in Vietnam' has been provisionally accepted for publication in PLOS Global Public Health.

Before your manuscript can be formally accepted you will need to complete some formatting changes, which you will receive in a follow up email. A member of our team will be in touch with a set of requests.

Please note that your manuscript will not be scheduled for publication until you have made the required changes, so a swift response is appreciated.

IMPORTANT: The editorial review process is now complete. PLOS will only permit corrections to spelling, formatting or significant scientific errors from this point onwards. Requests for major changes, or any which affect the scientific understanding of your work, will cause delays to the publication date of your manuscript.

If your institution or institutions have a press office, please notify them about your upcoming paper to help maximize its impact. If they'll be preparing press materials, please inform our press team as soon as possible -- no later than 48 hours after receiving the formal acceptance. Your manuscript will remain under strict press embargo until 2 pm Eastern Time on the date of publication. For more information, please contact globalpubhealth@plos.org.

Thank you again for supporting Open Access publishing; we are looking forward to publishing your work in PLOS Global Public Health.

Best regards,

Graeme Hoddinott, Ph.D

Academic Editor

PLOS Global Public Health

***********************************************************

Reviewer Comments (if any, and for reference):

Reviewer's Responses to Questions

**Comments to the Author**

1. If the authors have adequately addressed your comments raised in a previous round of review and you feel that this manuscript is now acceptable for publication, you may indicate that here to bypass the “Comments to the Author” section, enter your conflict of interest statement in the “Confidential to Editor” section, and submit your "Accept" recommendation.

Reviewer #1: All comments have been addressed

Reviewer #2: All comments have been addressed

Reviewer #4: All comments have been addressed

**********

2. Does this manuscript meet PLOS Global Public Health’s publication criteria? Is the manuscript technically sound, and do the data support the conclusions? The manuscript must describe methodologically and ethically rigorous research with conclusions that are appropriately drawn based on the data presented.

Reviewer #1: Yes

Reviewer #2: Yes

Reviewer #4: Yes

**********

3. Has the statistical analysis been performed appropriately and rigorously?

Reviewer #1: Yes

Reviewer #2: Yes

Reviewer #4: Yes

**********

4. Have the authors made all data underlying the findings in their manuscript fully available (please refer to the Data Availability Statement at the start of the manuscript PDF file)?

The PLOS Data policy requires authors to make all data underlying the findings described in their manuscript fully available without restriction, with rare exception. The data should be provided as part of the manuscript or its supporting information, or deposited to a public repository. For example, in addition to summary statistics, the data points behind means, medians and variance measures should be available. If there are restrictions on publicly sharing data—e.g. participant privacy or use of data from a third party—those must be specified.

Reviewer #1: Yes

Reviewer #2: Yes

Reviewer #4: Yes

**********

5. Is the manuscript presented in an intelligible fashion and written in standard English?

PLOS Global Public Health does not copyedit accepted manuscripts, so the language in submitted articles must be clear, correct, and unambiguous. Any typographical or grammatical errors should be corrected at revision, so please note any specific errors here.

Reviewer #1: Yes

Reviewer #2: Yes

Reviewer #4: Yes

**********

6. Review Comments to the Author

Please use the space provided to explain your answers to the questions above. You may also include additional comments for the author, including concerns about dual publication, research ethics, or publication ethics. (Please upload your review as an attachment if it exceeds 20,000 characters)

Reviewer #1: (No Response)

Reviewer #2: (No Response)

Reviewer #4: I have reviewed the revised manuscript and am satisfied that the authors have adequately addressed all of my concerns.

The spelling errors I flagged have been corrected and the authors indicate that a broader review of the manuscript for similar errors was undertaken.

The abstract now includes the adjusted odds ratios and confidence intervals that were previously missing, which improves the interpretability of the key findings.

The introduction has been substantially revised. The research gap is more clearly stated.

The sampling strategy in the methods section is now clearly described, with consecutive sampling for the quantitative component and purposive sampling for the qualitative component both explicitly stated.

The discussion has been meaningfully restructured. The authors have moved beyond descriptive reporting and now explicitly connect qualitative themes to quantitative associations.

The limitations section has been significantly expanded to address the epidemiological threats to validity I raised.

Cronbach's alpha for the stigma scale has been reported.

The recommendations section has also been rewritten and is now specific and actionable.

I am satisfied that my concerns have been adequately met and recommend the manuscript for further consideration.

**********

7. PLOS authors have the option to publish the peer review history of their article (what does this mean?). If published, this will include your full peer review and any attached files.

**Do you want your identity to be public for this peer review?**  If you choose “no”, your identity will remain anonymous but your review may still be made public.

For information about this choice, including consent withdrawal, please see our Privacy Policy.

Reviewer #1: **Yes:**  barış çil

Reviewer #2: No

Reviewer #4: No

**********
